# Open source framework for a Broadly Expandable and Reconfigurable data acquisition and automation device (BREAD)

**DOI:** 10.1016/j.ohx.2023.e00467

**Published:** 2023-08-25

**Authors:** Shane Oberloier, Nicholas G. Whisman, Finn Hafting, Joshua M. Pearce

**Affiliations:** a Department of Electrical & Computer Engineering, Michigan Technological University, Houghton MI 49931 USA; b Department of Electrical & Computer Engineering, Western University, London, ON, Canada

**Keywords:** Open Source, Data Acquisition, Automation Framework, Measurement Systems, Controls, Low Cost Alternative

## Abstract

Though open source data acquisition (DAQ) systems have been published, closed source proprietary systems are the standard despite often being prohibitively expensive. High costs, however, limit access to high-quality DAQ in low-resource settings. In many cases the functions executed by the closed source and proprietary DAQ cards could be carried out by an open source alternative; however, as desired function count increases, the simplicity of integrating the designs decreases substantially. Although the global library of open source electronic designs is expanding rapidly, and there is clear evidence they can reduce costs for scientists one device at a time, they are generally made to carry a function well, but are often not capable of scaling up or easily being integrated with other designs. Just as other open source projects have found success by having modular frameworks and clearly documented specifications, a framework to unify and enable interoperation of these open source electronics systems would be greatly beneficial to the scientific community. To meet these needs and ensure greater accessibility to high-quality electronics sensing and DAQ systems, this article shares and tests a news framework where new open source electronics can be developed and have plug-and-play functionality. The Broadly Reconfigurable and Expandable Automation Device (BREAD), consists of a basic set of guidelines and requirements to which others can contribute. Here 7 slices (boards) are provided, demonstrated, and validated: 1) Amplified Analog Input, 2) Audio Analysis / Fourier Transform, 3) +/- 10A Current Sensor, 4) 4-Channel Relay Controller 5) 4 Channel Stepper Motor Controller, 6) 4 Channel Type-K Thermocouple Reader and 7) 2 Channel USB Port. Implementing systems using BREAD rather than closed source and proprietary alternatives can result in cost savings of up to 93%.

Specifications table

**Table:** 

Hardware name	Broadly Reconfigurable and Expandable Automation Device (BREAD)
Subject area	- Engineering and materials science- General
Hardware type	- Imaging tools- Measuring physical properties and in-lab sensors- Field measurements and sensors- Electrical engineering and computer science- Mechanical engineering and materials science
Closest commercial analog	The National Instruments cDAQ platform
Open source license	GNU General Public License (GPL) 3.0
Cost of hardware	• SLC_LVAI Amplified Analog Input Slice: 40.61 USD • SLC_AAFT Audio Analysis / Fourier Transform: 32.64 USD • SLC_CR10 +/- 10A Current Sensor: 35.46 USD • SLC_RLAY 4 Channel Relay Controller: 62.01 USD • SLC_STEP 4 Channel Stepper Motor Controller: 50.57 USD • SLC_THRM 4 Channel Type-K Thermocouple Reader: 40.87 USD • SLC_USBP 2 Channel USB Port: 20.04 USD • LOAF_x08 8 slot loaf backplane: 13.37 USD • Total: 313.31 USD
Source file repository	https://osf.io/u2h4g/ DOI 10.17605/OSF.IO/U2H4G

## Hardware in context

The global library of open source electronic designs is constantly expanding and diversifying [1]. Examples include systems to accurately measure gas pressures [2] and properties [3]. There are devices used to aid in electrical engineering endeavours such as power monitoring [4] and phasor measurement [5] as well as complex fields such as neuroscience [6], [7], electrophoresis [8], and nuclear physics [9]. Platforms promoting and enabling citizen participation on environmental science have been prototyped [10], [11]. These devices are all made to carry out a handful of functions well but are often not capable of scaling up or easily being integrated with other designs. Just as other open source projects have found success by having modular frameworks and clearly documented specifications [1], [12], [13], [14], a framework to unify and enable communication between these open source electronics systems would be greatly beneficial to the scientific community. Such a framework would allow scientists and researchers to upgrade, expand, and reuse their control electronics to suit their needs in a variety of disciplines.

For example, a closed source, proprietary, fixed system designed to control four heaters in a material processing line greatly limits researchers. In this example, if a researcher wants to add another heater to preheat the material, they will have to buy another four-heater controllers and heater from the same company to ensure they are both compatible with the original system. In another case, the researcher may want to scale up the system to increase yield which would require more powerful heaters. Because the original system is fixed, new controllers and heaters would need to be purchased; dramatically increasing costs. If the researcher had an open source and modular control system instead, a new heater could be added with a single-heater controller. Because the system is open source, the original electronics could be modified by swapping out components for higher power alternatives to scale up the system. Open source DAQ systems like Fieldkit provide users with modular, plug-and-play hardware for environmental sensing [15]. Additional hardware is required, however, when researchers want to automate an experiment by controlling, for example, a valve, heater, motor, or other component. A framework which integrates inexpensive DAQ hardware with supervisory control would greatly benefit researchers. Also, by having precise control over experimental variables, researchers can easily improve the repeatability and reliability of their experiments.

Though open source data acquisition (DAQ) systems have been published [16], [17], [18], National Instruments (closed source, proprietary) systems are used extensively in academia for diverse purposes ranging from analysis of agricultural tools [19] and snow analysis [20] to CNC systems [21] and battery research [22]. National Instruments CompactDAQ (cDAQ) [23] systems are often chosen for their flexibility, modular design, and plug-and play operation. The cost of such systems and those from their closed source and proprietary competitors can be prohibitively expensive, ranging around $1,000 USD for a chassis and anywhere between $138 USD to $2,846 USD per function card [23]. These costs limit access to high-quality DAQ in low resource settings [24], separating haves and have nots [25]. In many cases the functions executed by the cDAQ cards could be carried out by an open source alternative, however as desired function count increases, the simplicity of integrating the designs decreases substantially.

To overcome these challenges and ensure greater accessibility to high-quality electronics sensing and DAQ systems, this manuscript proposes a framework where new open source electronics can be developed and have plug-and-play functionality with all other modules designed in this framework. The system, titled *Broadly Reconfigurable and Expandable Automation Device (BREAD)*, consists of a basic set of guidelines and requirements to which others can contribute.

The six function cards explored in this paper have similar counterparts to National Instruments’ CompactRIO (cRIO) system and are compared in their functionality and cost. While the cRIO system is designed to work with other industrial automation devices, BREAD is designed to be an “all-in-one” system for DAQ, sensing, and supervisory control applications. On average, the costs savings of using BREAD over the cRIO system is 93% when comparing the six function cards.

## Hardware description

BREAD first and foremost is set of specifications with intent to unify open source electronic designs as an open source DAQ and automation platform, and as such the focus of this manuscript will be on the generalized guidelines. Several child cards (referred to as *slices*), and a parent card (referred to as a *loaf*) have been developed and will also be described.

### Configuring a system

BREAD can be used in three different configurations: 1) in single slice mode, 2) single loaf mode, or 3) multi-loaf mode.

#### Single slice usage

**Fig. 1:**
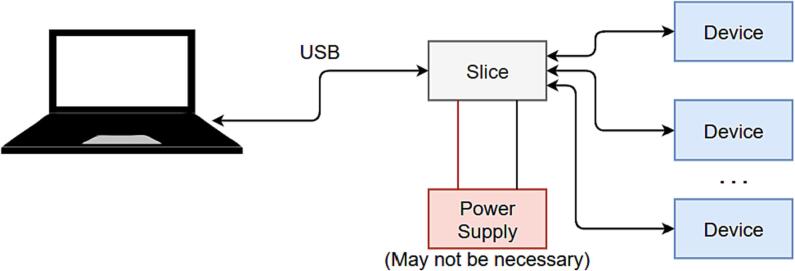
Schematic of single slice mode operation.

In single slice mode, the slice is used as its own leader; receiving and sending data to the connected computer as shown in Fig. 1. In some (depending on the requirements of the card), an external power supply may be required.

#### Single loaf usage

**Fig. 2:**
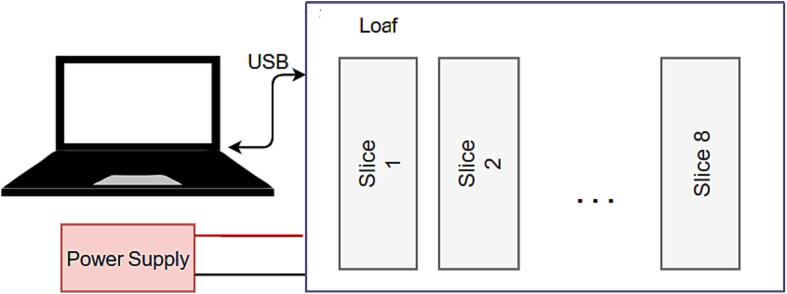
Schematic of single loaf mode operation.

In single loaf mode the loaf acts as leader to multiple slices as shown in Fig. 2. Power to each slice is distributed via the loaf, as well as critical signals such as e-stop and sync. In this mode slices may still be controlled via serial or I2C, which is useful for diagnostics.

#### Multi-Loaf usage

**Fig. 3:**
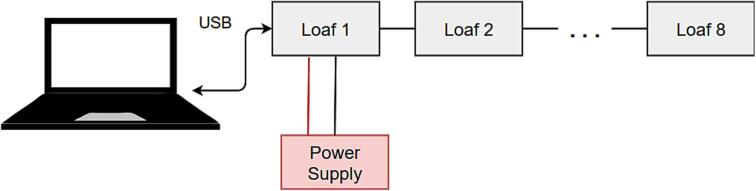
Schematic of multi-loaf mode operation.

Loafs are designed with a connection point by way of a three-wire connector to extend I2C communication. Using these connectors, multiple loafs (up to 8 loafs, totalling 64 possible slices) can be daisy-chained as shown in Fig. 3. Depending on power requirements each loaf may need a separate power supply.

### Example applications

As the number of unique Slices increases, the applications for BREAD will also increase. Slices capable of temperature monitoring, pH monitoring, dissolved oxygen monitoring, heating control, and motor control, for example, could be used to automate a variety of material processing systems like a stirred tank bioreactor [26]. In another case, scientists may want a DAQ system to capture environmental data in the field like temperature, humidity, pressure, and air quality. BREAD could be deployed in this setting as well. Due to the modular nature of BREAD, smaller scale operations, like monitoring soil moisture content, can also be accomplished with a single Slice. While open source DAQ systems exist, BREAD expands this domain by incorporating supervisory control; providing scientists, makers, and DIY engineers with the tools to tackle a wide range of experiments, projects, and automation solutions. In situations where a researcher who builds their own hardware needs a specific circuit to meet their needs, they could modify an existing Slice design. In this case, having many Slices with robust electrical designs would reduce duplicated effort by providing a starting point for new electrical designs.

### Example System: TIG-Based metal 3D printer Monitor

As with many processes, data acquisition can be an important component of characterizing novel rapid prototyping equipment [17]. In this manuscript, a DAQ system utilizing BREAD componentry will be proposed and constructed for a metal 3-D printer. Metal 3-D printing has not become as ubiquitous as polymer 3-D printing, partially because there are no low-cost, closed source, and proprietary options available [27]. Lowering the barrier to entry for digital metal manufacturing may allow engineers and hobbyists alike to create and share metal components in their designs.

#### Required functions & slices

Taking inspiration from traditional welding, the most important feedback to consider is light (intensity and modulation) and sound [28]. These signals generated by the arc can be used to indicate variables like improper currents, gas flow, feed rate, and stand-off distances [17]. Light can be measured using a photoresistor paired with the SLC_LVAI slice. Sound can additionally be measured via the SLC_LVAI slice.

In addition, the BREAD system will be used to take power measurements using a non-invasive current transformer placed on the return line. The sensor can also be measured using the SLC_LVAI slice. Finally, a SLC_THRM can be used to measure the temperature of 4 corners of the print substrate.

#### Configuration

**Fig. 4:**
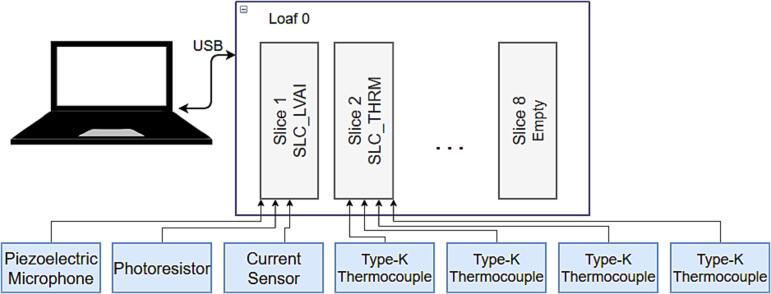
An example BREAD configuration for monitoring the quality of a welding process.

The system is built using a LOAF_X08 8 slice backplane. The connection diagram is shown in Fig. 4. The microphone and photoresistor are placed on the end-effector carriage to keep a consistent reference frame for measurement. The current sensor is placed around the ground wire of the welder. A type K thermocouple is mounted at each corner of the printing substrate.

#### Firmware

**Fig. 5:**
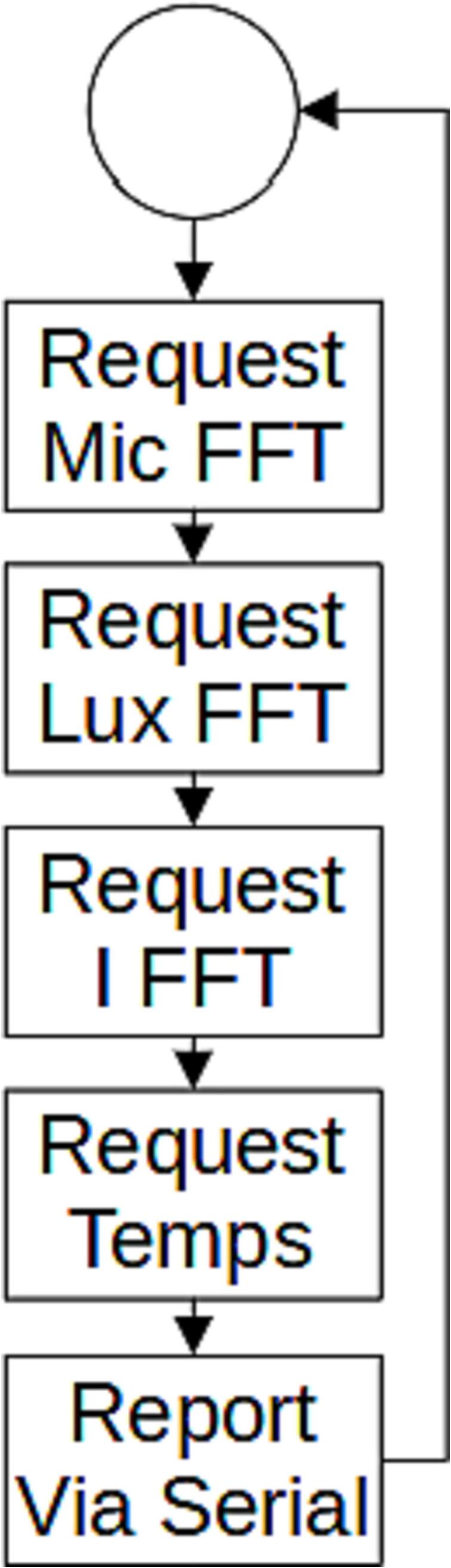
The weld monitoring sample loop.

The firmware for this example system consists of a sample-record-repeat structure (Fig. 5). The routine is set to sample at a frequency of 5 Hz, with each sample being timestamped by the Arduino’s system clock. The SLC_LVAI slice is set to capture an FFT on each sensor.

#### Schematic design procedures for slices

First, the schematic standards will be laid out. Though many of the standards in this section do not affect the final design, proper and consistent documentation is crucial to the success of open source hardware projects [29], [30].

#### Starting out the design

Every BREAD slice board design should be duplicated from the template slice, SLC-TEMP. This ensures that the mechanical dimensions of the board are correct, as well as the interface pin assignment. The design files can be modified using the most recent version the open source KiCAD software [31].

#### Design standards & conventions

##### Standard components & connections

The standard components in the template design (included in SLC-TEMP) are essential for communication, computation, and measurement, and therefore should not be modified or moved in any way. Additionally, they should not be directly connected to, instead each connection should be made using net labels. The “bus” wiring functionality should only be used if 5 or more highly coupled signals are present.

The standard components provided in the template include an Arduino Nano, interface connector, and power filtering capacitors. Together these components provide a robust system for commutation, measurement, and control.

##### Component selection

When possible, all discrete components should be of a 1206 (3.2 mm × 1.6 mm footprint) package or greater. Only commonly available decade values should be used for discrete components such as resistors and capacitors. When selecting specialized components (i.e. Application Specific Integrated Circuits or ASICs), select the package that is easiest to be soldered (if possible, select through hole, socket-able chips).

##### Sub-Circuits

All designs should be split into sub-circuits. In most cases, each ASIC, filter, supply, conditioner, etc. should be their own sub-circuit. If a sub-circuit is repeated more than twice, it should be copied into a hierarchical sheet and replicated. All sub-circuits should interconnect using net labels.

##### Aesthetics, Labelling, and comments

All net routing should be made such that there is minimal visual cross-over, and nets should be spread apart when possible. Though it is not required – all nets can be labelled. All sub-circuits should be surrounded by a graphical box with a title at the top. If enough space is available, a short description of the sub-circuit should be included. Labelling and terminology should be non-specific so sub-circuits can seamlessly be copied to new designs. All jumpers must be clearly labelled, as well as their default state. A jumper configuration table is recommended as shown in Table 1.

**Table 1:** An example jumper configuration table.

Jumper ID	Pin 0 & 1 Bridged	Pin 1 & 2 Bridged
J0	Utilize External Voltage Source	Utilize Internal Voltage Source
J1	AC Measurement Mode	DC Measurement Mode

##### Annotation & Net-List generation

When annotating the schematics (generating component numbers and names) select the “First free after sheet number x100” option. This will help especially in designs which use hierarchical sheets. The standard components may be renamed. Generated netlists should have the same name as the.pro file.

### Board design procedures for slices

#### Starting out the design

The layout (kicad_pcb) file is included with the template design slice (included in SLC-TEMP). It is highly recommended that this layout be adapted for all designs for the sake of a consistent library of slices.

#### Considerations for digital manufacturing

As BREAD is an open source system geared toward DIY engineers, scientists, and makers, it is highly desirable that the boards are manufacturable on commonly available circuit milling machines [32], [33]. Designs that meet these criteria should:

• Have routing all done on a single layer (on the bottom layer)
• All surface-mount components should be flipped to the bottom layer
• All through-hole components should remain on the top layer
• Minimum trace size: 0.5 mm, minimum feature spacing: 0.2 mm, pad copper width greater than 0.5 mm
• When trace cross-over is unavoidable use the second layer, but also use vias such that a fine-gauge wire can be substituted as a bridge.
• Do not use planes if possible – they can cause redundant milling for some CAM software packages.

It is also recognized that some boards cannot conform to these standards due to required parts, complexity, etc. In these cases, it is allowed. The goal of BREAD is to be an inclusive family of components, but the ability to digitally manufacture most boards is something that can be valuable to BREAD’s overall success [29].

#### Design standards & conventions

##### Component placement

When placing components, take into consideration that the boards may be assembled by inexperienced solderers. Parts should be placed with clearance between one another to make accidental solder-bridging less likely. Additionally, components should be grouped with other components from their sub-circuit. All large components (likely through-hole) should stay on the top of the board. Devices that may generate heat should be given suitable clearance to dissipate the heat.

##### Routing

To reiterate a previously mention standard: If the board is a single layer - when trace cross-over is unavoidable use the second layer, but also use vias such that a fine-gauge wire can be substituted.

If the board is dual layer – the top layer’s traces should be predominately vertical, and the bottom layer’s traces should be predominately horizontal. If possible, use large enough vias (1 mm) with pads around them so that any users that can mill two-sided boards can use a thin wire to connect both sides of the via. If the board is more than 2 layers – it is outside of the range of the vast majority of DIYers, and thus the board will have to be manufactured professionally. In this case, considerations for PCB millers can be removed. Traces should be appropriately sized for their expected currents.

##### Planes

In most cases, planes are used for power distribution. For two-layer boards, the ground should be on the top layer, and the dominant supply voltage should be the bottom layer. Designers should be sure to include proper thermal reliefs for any connections to the plane.

##### Labelling

All device designators should be in the same orientation and not covered by any devices. Jumpers should have indicators on what each one controls. All test points should have their corresponding net names labelling them. Each pin on the outgoing connector should have a clear label, as well as any indicator LEDs. The interface connector does not need labelled.

The design should have a board designator with the following information:

• Card Name
• Part #
• Rev # and year

**Fig. 6:**
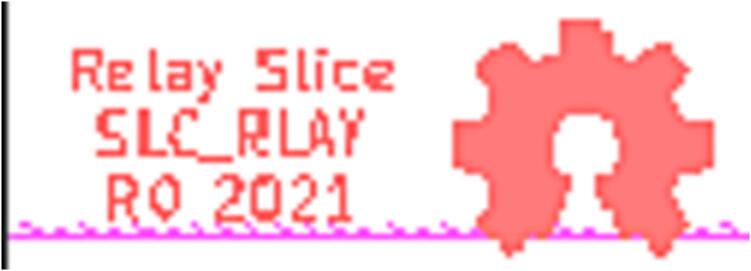
An example board designator and OSH logo.

Finally, the Open Source Hardware (OSH) logo should be visible somewhere on the board (preferably near the board designator). An example of a board designator is shown below in Fig. 6.

#### Gerber generation

When generating gerbers, be sure that the grid origin and the drill and place offset are both at the bottom left corner of the board. Be sure to select “use auxiliary axis as origin” and generate the F.Cu, B.Cu, F.Paste, B.Paste, F.SilkS, B.SilkS, F.Mask, B.Mask, and Edge.Cuts layers. Additionally, generate all drill files and be sure to use the auxiliary axis as the drill origin. Having a properly placed auxiliary axis is helpful to those assembling with pick-and-place machines.

### Documentation procedures for slices

#### Standard datasheet

BREAD_SLC_TEMP_R0.odt is provided with the SLC_TEMP repository and should be filled out according to the instructions embedded in the document. With each revision of the circuit, the document should be revisited (and the revision section should have notes on what was changed).

#### Repository

Though it is not required, it is highly recommended that the slice documentation and design files are hosted in an Open Science Framework (OSF) repository [34]. The repository should be organized by dividing it into 5 folders:

• Documentation
• PCB Design
• Firmware
• Gerbers
• Mechanical

### Mechanical definitions for slices

#### PCB dimensions

**Fig. 7:**
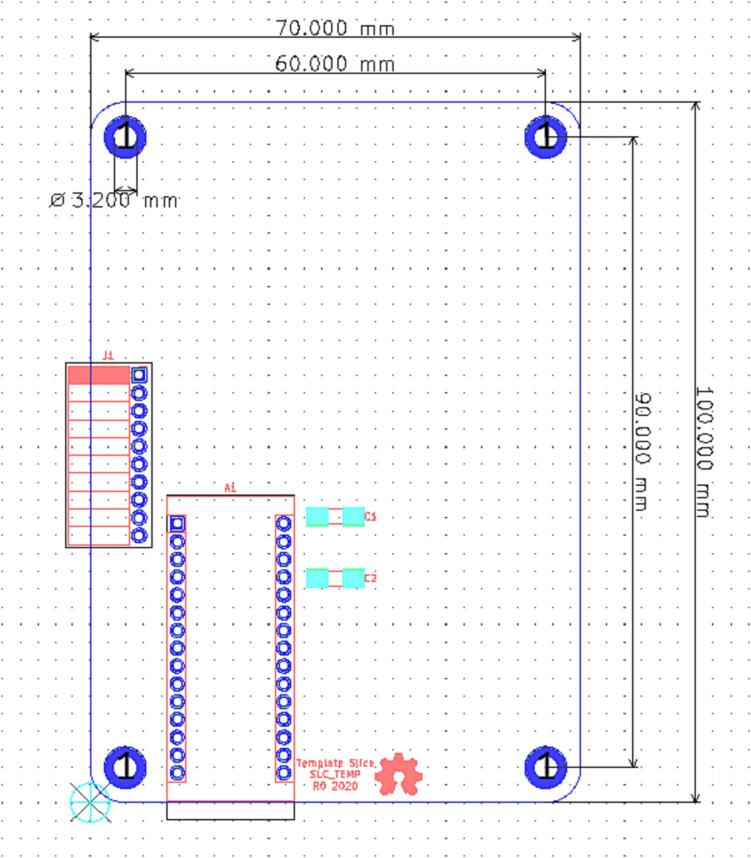
The Mechanical specifications of a slice PCB.

Slices are constructed on an 100 mm by 70 mm board with 4 mounting holes for am M3 bolt inset 5 mm into the board at each corner (See Fig. 7).

#### Positioning & sizing constraints

**Fig. 8:**
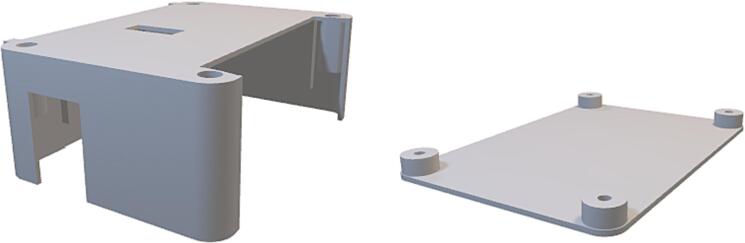
A rendering of the default enclosure for a slice.

For a standard slice, the Arduino Nano [13], interface connector, mounting holes, and board dimensions should remain consistent with SLC_TEMP. Doing so will ensure compatibility with the default enclosure. The default enclosure for a slice is shown in Fig. 8.

### Loaf backplane

**Fig. 9:**
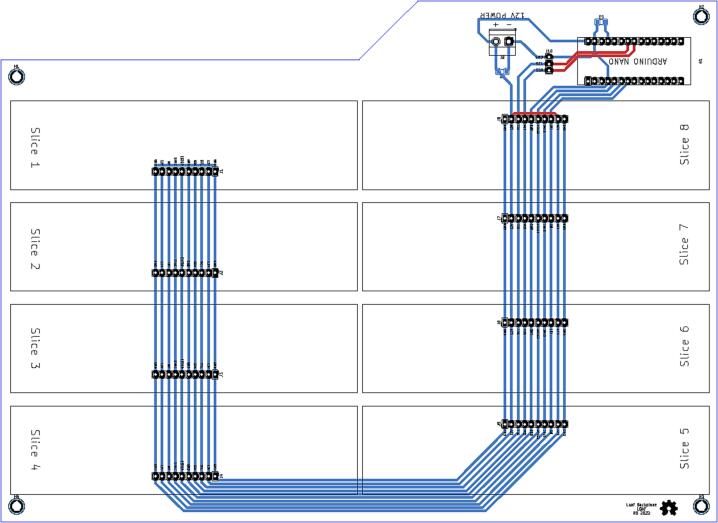
Routed circuit board for the Loaf backplane.

**Fig. 10:**
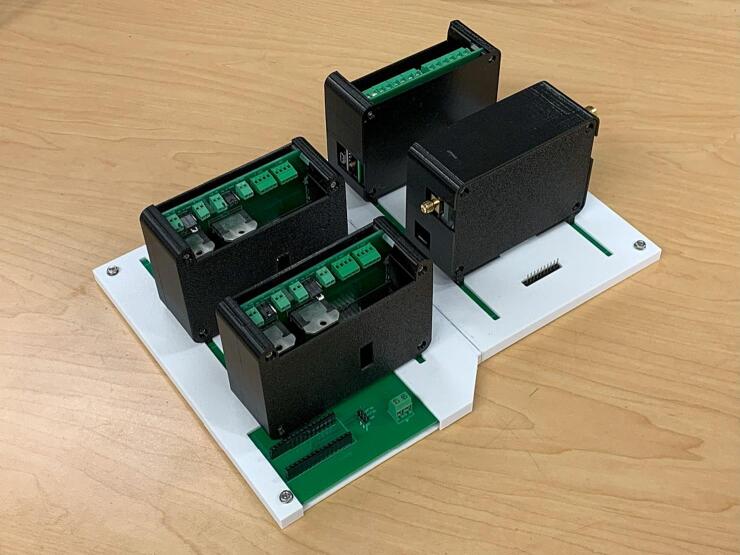
Example system with four Slices connected to a Loaf.

To enable communication and power delivery to each Slice, a parent board called a “Loaf” was developed. The electrical schematic for the loaf backplane is shown in Fig. 9. The loaf backplane consists of eight 10-pin headers for connecting slices, a terminal block for connecting 12 V external power, and a 3-pin connector for multi-loaf functions. Each Slice plugs into one of the 10-pin headers (Fig. 10).

### Electrical definitions for slices

The electrical specifications for a slice assume the use of an Arduino Nano per SLC_TEMP. If resources permit, a Nano is recommended, however other controllers can be adapted to follow the BREAD protocols. The Arduino Nano is used as the default microcontroller platform due to low cost, high availability, and the use of an open source framework [13].

#### Loaf interface connector

**Fig. 11:**
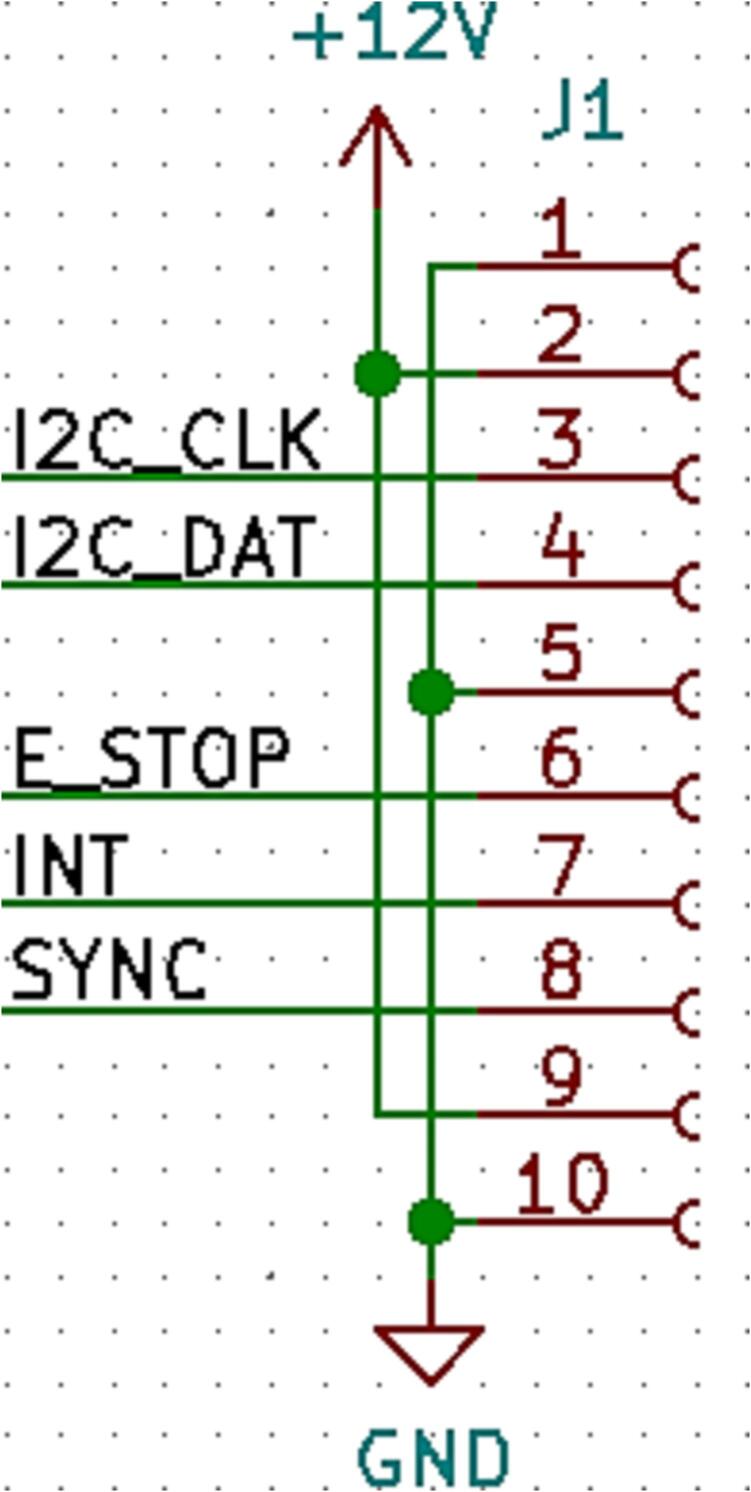
A schematic of the loaf interface connector.

Each slice should have a standard 10-pin female header centred on the left side (opposite to the external connector) of the PCB. The pin breakout is shown in Table 2 and a schematic is shown in Fig. 11.

**Table 2:** Pin breakout names and descriptions.

**Pin #**	**Name**	**Description**
1	GND	System ground and reference
2	+12 V	Voltage from loaf supply
3	I2C_CLK	Clock signal for communication between the slice and loaf
4	I2C_DAT	Data signal for communication between the slice and loaf
5	GND	System ground and reference
6	E_STOP	Signal (driven by loaf) which goes through physical interlocks acting as an E-Stop
7	INT	Interrupt pin (driven by slice) used to indicate to loaf that special attention is needed
8	SYNC	Digital signal (driven by loaf) used to synchronize actions across multiple slices
9	+12 V	Voltage from loaf supply
10	GND	System ground and reference

#### External connector

The most commonly used external connector (to devices and sensors) is the 2.54 mm (0.1″) pitch screw terminal. In some cases, however, specialized connectors may be needed. If possible, the connector should be a plug for a cable-end jack for ease of wiring.

#### Default circuitry & connections

**Fig. 12:**
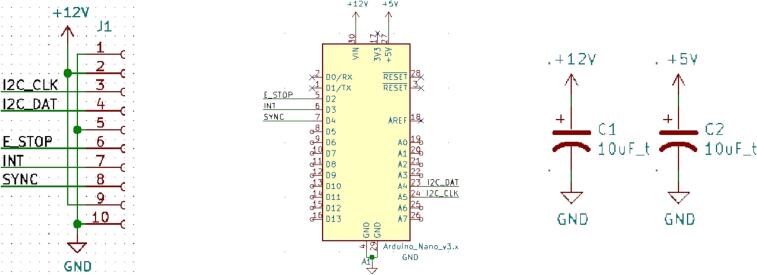
The default components used on every standard slice.

Though substitutions are allowed, SLC_TEMP utilizes an Arduino Nano for processing, measurement, computation, and communication. Additionally, the Arduino should have 10uF capacitors on both the 5 V and 12 V line. Every slice must have the loaf interface connector. The default components used on every standard slice are shown in Fig. 12.

### Software definitions for slices

For BREAD to be a cohesive system, documentation, including code, must also follow a set of guidelines.

#### Programming standards

All firmware for the Arduino-based slices and loafs should be programmed in the Arduino Integrated Development Environment (IDE) and follow the style guide as laid out by Arduino [35]. All pin names should be consistent with their names as represented on schematics.

### Existing slices

At the time of publishing there are 7 available slices. Their titles are:

1. SLC_LVAI: Amplified Analog Input Slice [36]
2. SLC_AAFT: Audio Analysis / Fourier Transform [37]
3. SLC_CR10: +/- 10A Current Sensor [38]
4. SLC_RLAY: 4 Channel Relay Controller [39]
5. SLC_STEP: 4 Channel Stepper Motor Controller [40]
6. SLC_THRM: 4 Channel Type-K Thermocouple Reader [41]
7. SLC_USBP: 2 Channel USB Port [42]

The slice functions and characterization procedures are listed in the following sections, with additional information available in each slice’s respective repository.

#### SLC_LVAI Amplified Analog input slice

**Fig. 13:**
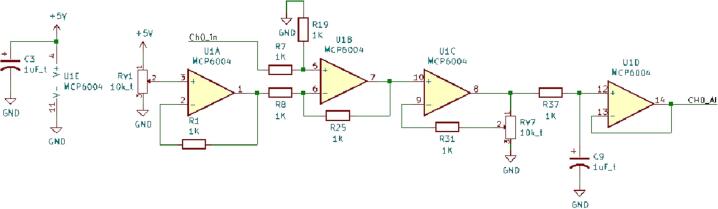
The offset, amplify, and filter circuit for channel 0 (typical for all channels).

SLC_LVAI has 6 adjustable analog measurement channels based around the MCP6004 [43] 4-channel operational amplifier. Two potentiometers are available to adjust offset and gain, theoretically allowing for a signal offset of 5 V and a gain of 11. Additionally, a first-order low pass filter is implemented with a corner frequency of 160 Hz (component can be replaced to alter cut off frequency) (Fig. 13).

**Fig. 14:**
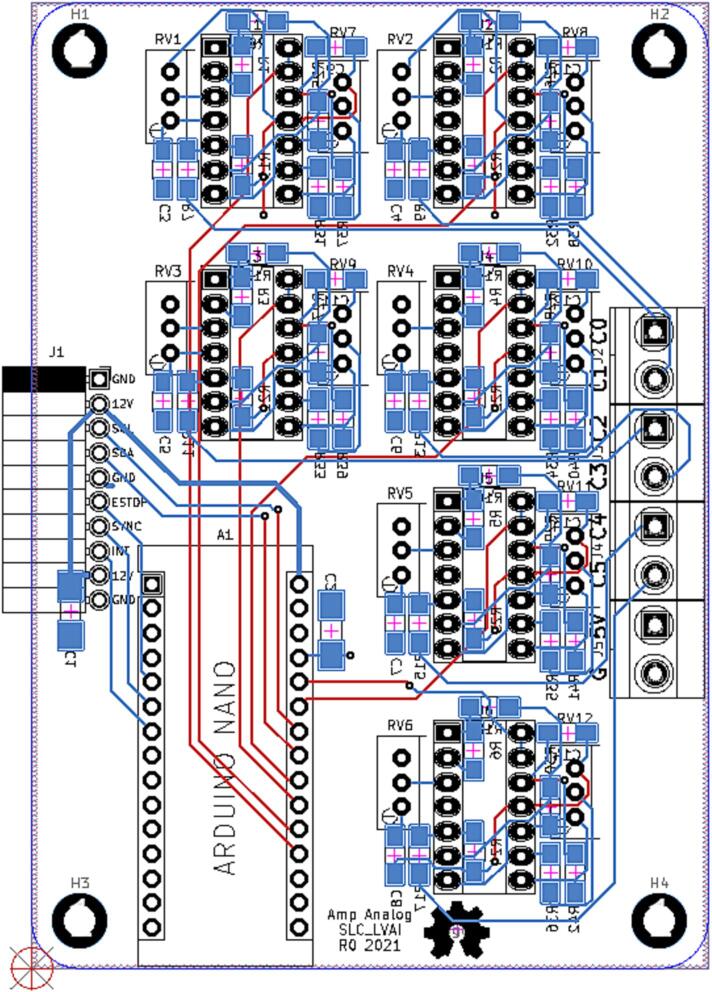
The routed circuit board for SLC_LVAI.

SLC_LVAI can be used to interface with a multitude of small-signal voltage sensors such as light sensors [44], sound sensors [45], and pressure sensors [46]. This slice could be used for applications such as solar tracking [47], automated chemical processing [48], and power measurement [4]. The circuit board layout for the slice is shown in Fig. 14.

The following functionality must be validated for the SLC_LVAI:

• Accurate reading of input analog signals.
• Ability to read in external commands and adjust accordingly.
• Ability to export data quickly and accurately.
• Ability to operate indefinitely and predictably.

Accurate Analog Readings.

1. Input a known, measured signal into a channel of SLC_LVAI.

2. View this signal on both an oscilloscope and the serial monitor of the Arduino IDE.

3. Tweak Potentiometers until signal appears to be correct shape.

4. Repeat process for every channel of SLC_LVAI.

Reading Commands.

For slice-only use:

1. Connect Slice to personal computer and open the Arduino IDE.

2. Open the Serial Monitor and attempt to transmit a command in proper form.

3. Verify the system responds properly to all commands that are sent to it. (Peripheral devices may need to be connected to see effects).

For system use:

1. Insert Slice into back plane.

2. Command the back plane to send a command to the slice.

3. Verify the system responds properly to all commands that are sent to it. (Peripheral devices may need to be connected to see effects).

#### SLC_AAFT Audio analysis / Fourier Transform

**Fig. 15:**
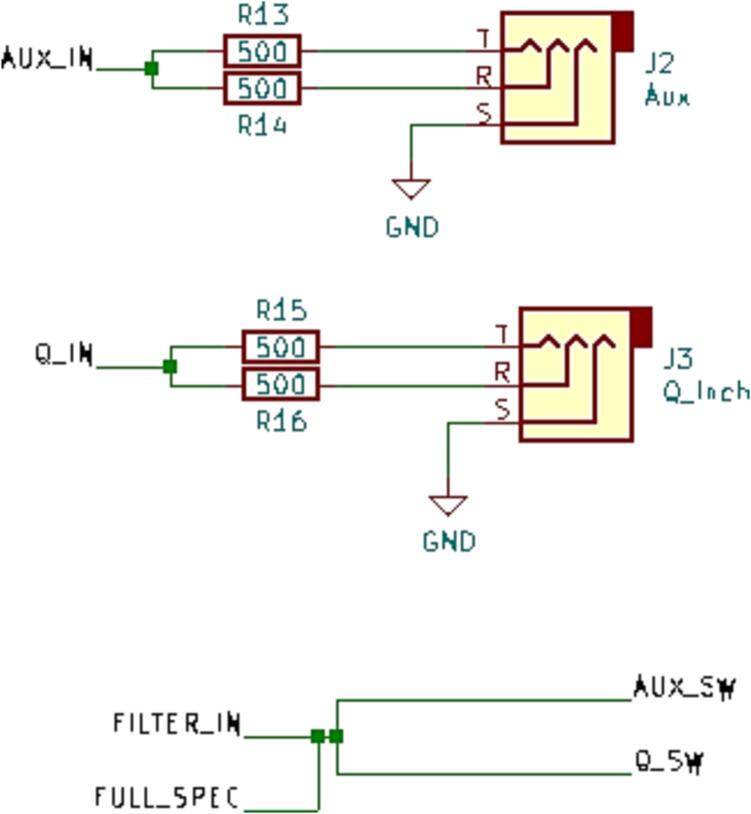
Channel Inputs and Signal Bridge.

**Fig. 16:**
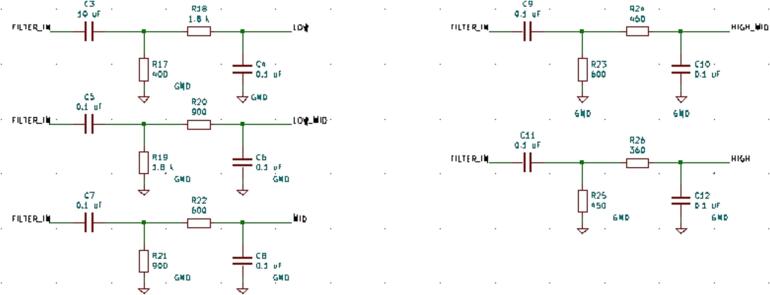
Frequency Breakdown Filters.

**Fig. 17:**
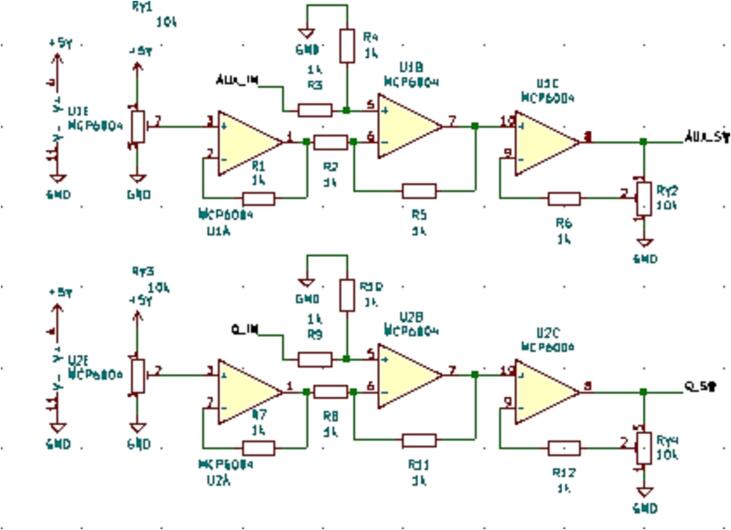
4 Stage Amplifier for Each Channel.

**Fig. 18:**
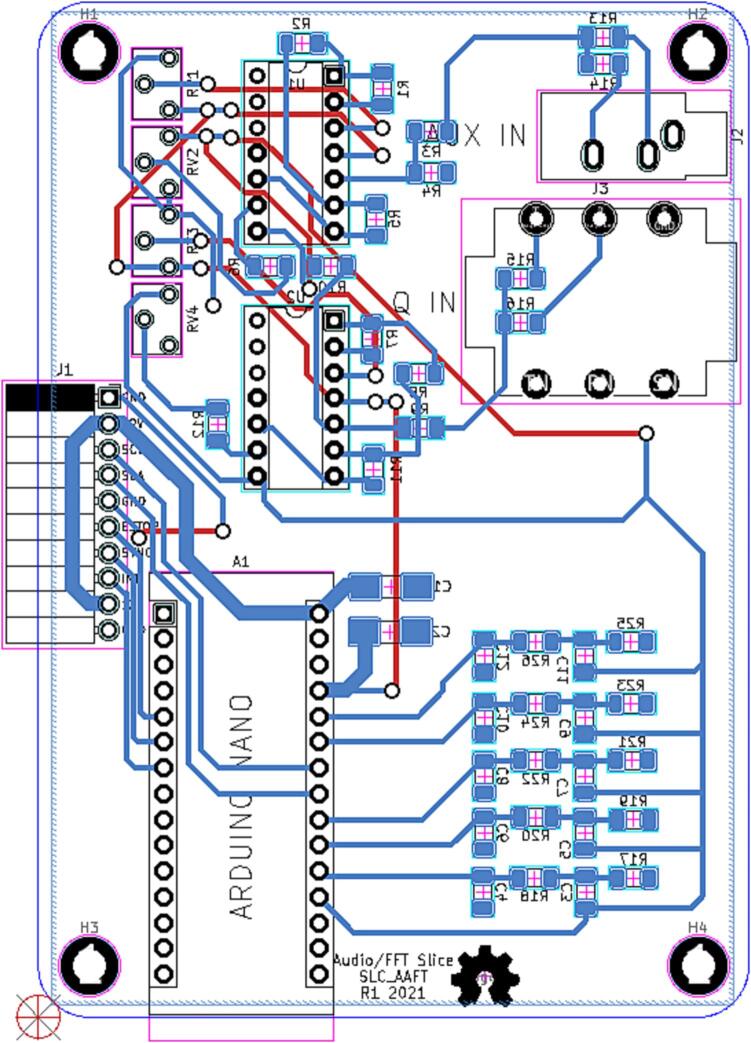
The routed circuit board for SLC_AAFT.

SLC_AAFT consists of two separate audio channels-- a quarter inch jack input and a standard aux input (Fig. 15). The input analog signal is broken down into low, low-mid, mid, high-mid, and high ranges, bounded between ∼ 0–5000 Hz (Fig. 16). The signal can be offset and amplified using a circuit based around the based around the MCP6004 [43] 4-channel operational amplifier (Fig. 17). Potential applications include analysis of system acoustics [49], musical audio analysis [50], and peak finder [51]. This slice is designed specifically to be connected with microphones (either for scientific measurement [52] or entertainment [53]) and musical instruments [54]. The circuit board layout for the slice is shown in Fig. 18.

The following functionality must be validated for the SLC_AAFT:

• Accuracy of board’s Fast Fourier Transform (FFT).
• Ability to send FFT data quickly and accurately to separate module.
• Ability to take in external signal data and run an FFT on it.
• Ability to operate indefinitely and predictably.

FFT Accuracy.

1. Feed a signal of known frequency composition into SLC_AAFT via one of the two channels.

2. View this signal on an oscilloscope concurrently.

3. Set the oscilloscope to output a real-time FFT of the input signal.

4. View SLC_AAFT’s generated FFT in the serial plotter window of the Arduino IDE.

5. Cross compare. Ensure no major discrepancies.

6. Repeat for the additional channel.

FFT Data Transfer.

1. Have SLC_AAFT run an FFT on a signal of known frequency composition.

2. Observe FFT results in Serial Monitor. Record.

3. Command separate device to read in entirety of FFT data. Observe and record.

4. Ensure no discrepancies.

External Signal FFT.

1. Use another module (such as SLC_LVAI) to read and send a known signal to SLC_AAFT via.

I2C.

2. View the signal being read by said separate module on an oscilloscope.

3. Configure the oscilloscope to output a real-time FFT of this test signal.

4. Command SLC_AAFT to run an FFT on this data.

5. Cross compare the two FFTs and ensure no discrepancies.

## Indefinite / Predictability test

1. Input a known signal into SLC_AAFT via either input channel.

2. Have SLC_AAFT continuously run an FFT on the data.

3. Allow to run for an arbitrarily long amount of time.

4. Ensure that the system is still producing results as expected at the end of said time period.

### SLC_CR10 +/- 10A current sensor

**Fig. 19:**
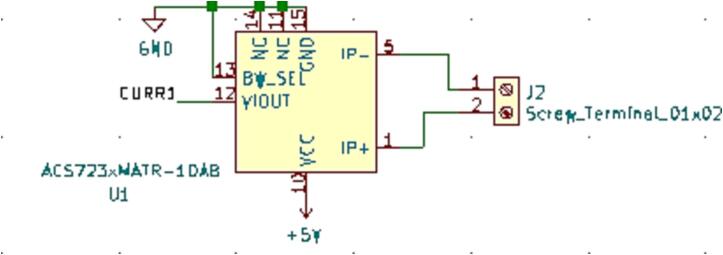
Typical sensor chip implementation for all channels.

**Fig. 20:**
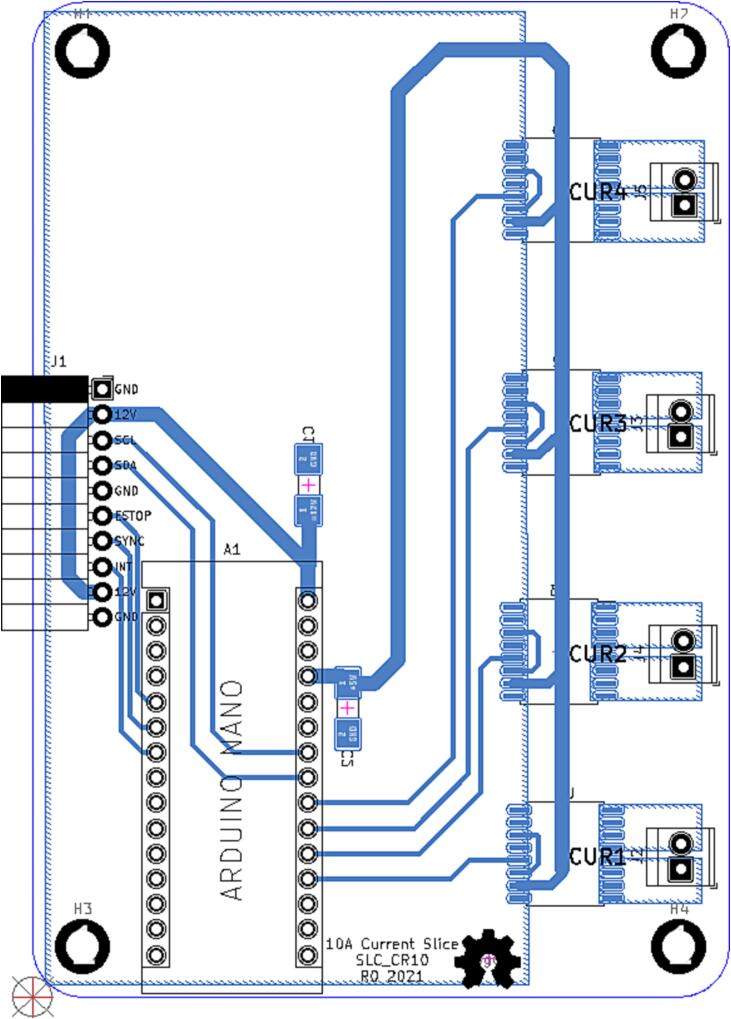
The routed circuit board for SLC_CR10.

SLC_CR10 consists of four individual current sensing channels based around the ACS723 Hall effect current sensor [55]. Current is routed through a hall effect current sensing chip, which then generates an analog signal to the Arduino (Fig. 19). Potential applications include power analysis of known system [4] signal detector [56], and automatic surge [57] alert system. The circuit board layout for the slice is shown in Fig. 20.

The following functionality must be validated for the SLC_CR10:

• Ability to read in external commands and adjust accordingly.
• Accuracy of input current signal on all channels.
• Ability to export data quickly and accurately.
• Ability to operate indefinitely and predictably.

Procedure.

Current Signal Read.

Set up a simple DC circuit with a known current, and hook SLC_CR(10/20/40) in series with it.
Verify the current read by the Slice is accurate.
Repeat with a different known current. Be sure to test range extremities.
Set up an AC circuit where the current signal is known / easily calculable.
Take current readings with SLC_CR(10/20/40). Plot the output data and ensure that the signal is being correctly read.
Repeat with a different known current signal. Be sure to test range extremities.
Repeat the above experiments on each channel.

Reading Commands.

For slice-only use:

Connect Slice to personal computer and open the Arduino IDE.
Open the Serial Monitor and attempt to transmit a command in proper form.
Verify the system responds properly to all commands that are sent to it. (Peripheral devices may need to be connected to see effects).

For system use:

Insert Slice into back plane.
Command the back plane to send a command to the slice.
Verify the system responds properly to all commands that are sent to it. (Peripheral devices may need to be connected to see effects).

Signal Data Transfer.

Issue a command to SLC_CR(10/20/40) to send current data for any given channel or mix of channels.
Record the sent data from the Slice.
Cross-compare with the received data. Ensure no discrepancies.

Indefinite / Predictability Test.

Command the Slice to read current on one (or many) channel(s).
Read the current signals on the Serial Monitor.
Allow the readings to persist for an arbitrarily long amount of time.
Ensure, after the proper amount of time has passed, the system is still operating as expected.

### SLC_RLAY 4 Channel Relay Controller

**Fig. 21:**
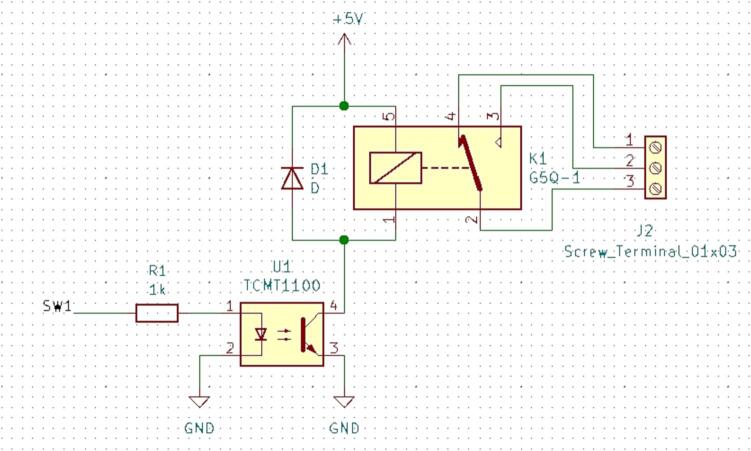
Common implementation of relay control channels.

**Fig. 22:**
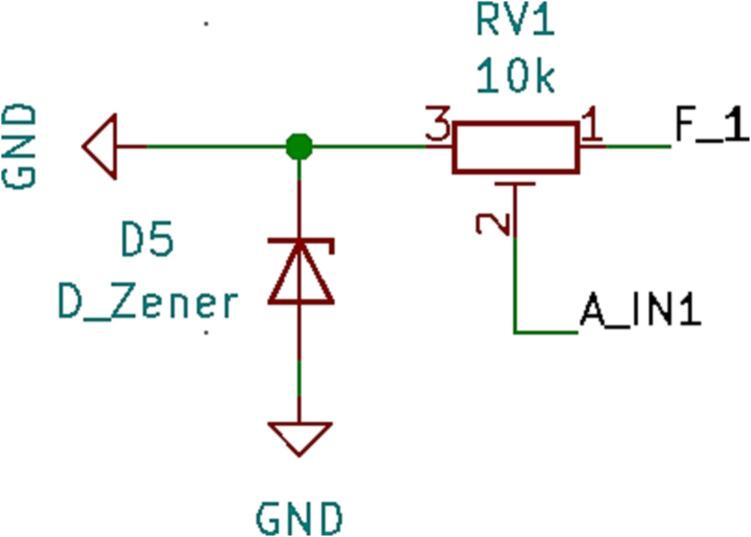
Analog feedback path.

**Fig. 23:**
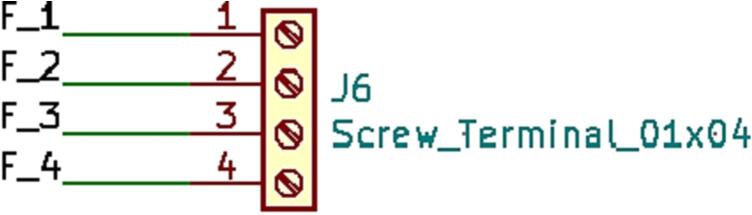
Feedback inputs.

**Fig. 24:**
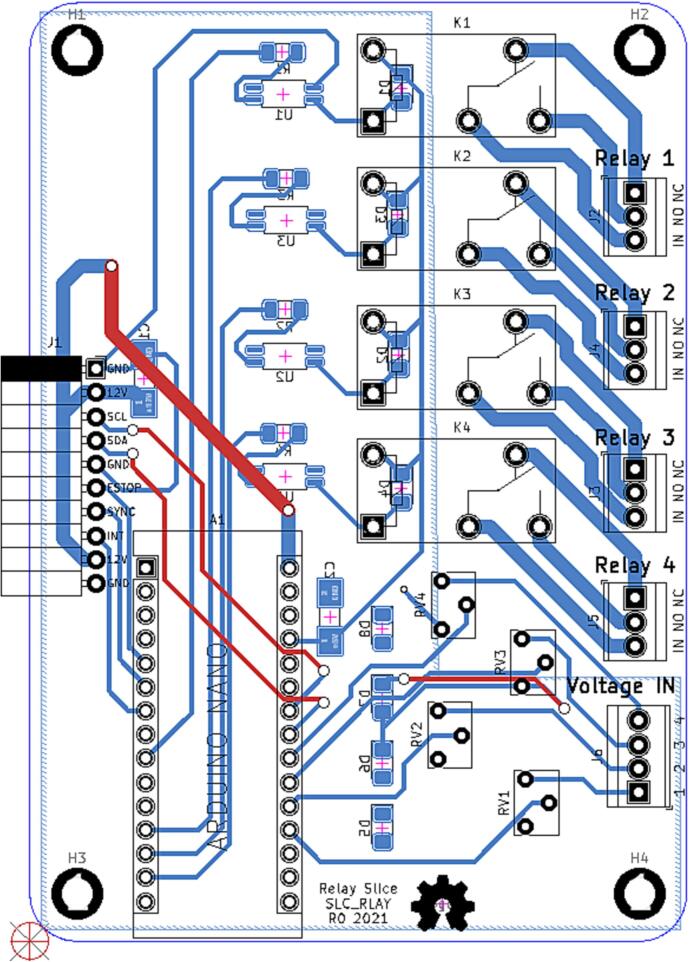
The routed circuit board for SLC_RLAY.

SLC_RLAY consists of 4 different relay control channels. Each channel is digitally controlled and opto-electrically isolated to allow for backwards voltage protection (Fig. 21). Four analog input channels are provided as a potential feedback / sensing path (Fig. 22, Fig. 23). The potentiometer on each of these paths serves to manually scale the input to a desired amplitude. Potential applications include automatic signal switching and timing of power supplies. The circuit board layout for the slice is shown in Fig. 24.

The following functionality must be validated for the SLC_RLAY:

• Accuracy
• Ability to read in external commands and adjust accordingly.
• Ability to export data quickly and accurately.
• Ability to operate indefinitely and predictably.

Procedure.

Reading Commands.

For slice-only use:

Connect Slice to personal computer and open the Arduino IDE.
Open the Serial Monitor and attempt to transmit a command in proper form.
Verify the system responds properly to all commands that are sent to it. (Peripheral devices may need to be connected to see effects).

For system use:

Insert Slice into back plane.
Command the back plane to send a command to the slice.
Verify the system responds properly to all commands that are sent to it. (Peripheral devices may need to be connected to see effects).

Exporting Data.

Indefinite / Predictability Test.

### SLC_STEP 4 Channel Stepper motor Controller

**Fig. 25:**
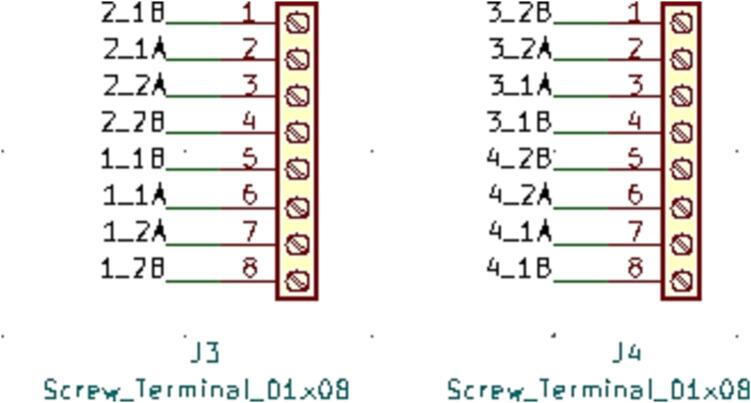
Motor output paths.

**Fig. 26:**
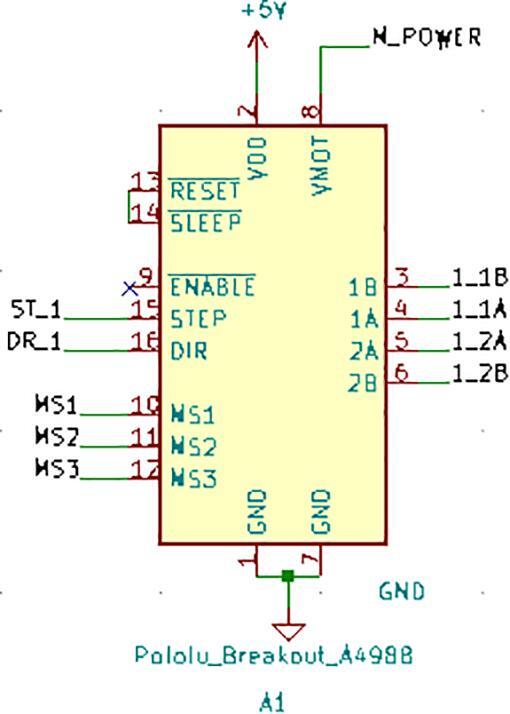
Stepper driver configuration for each channel.

**Fig. 27:**
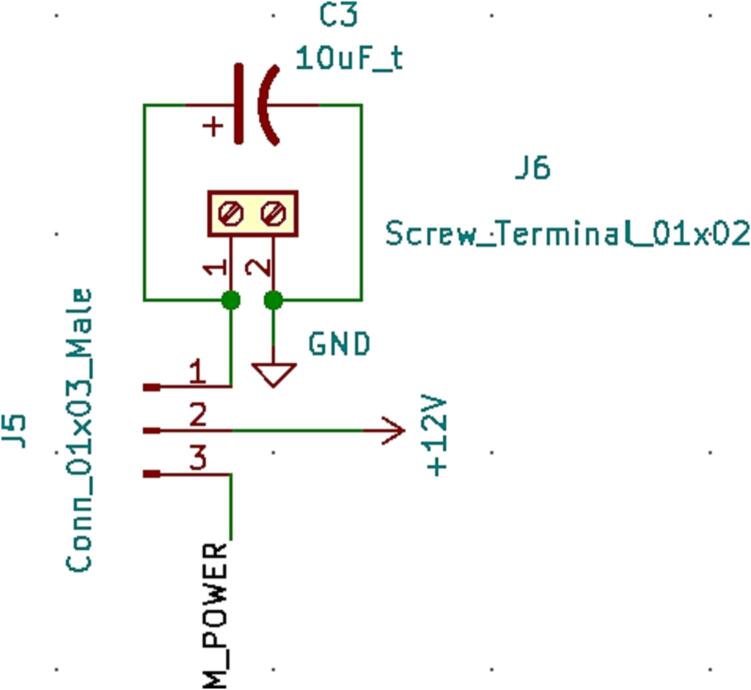
Custom Power Supply Input + Select.

**Fig. 28:**
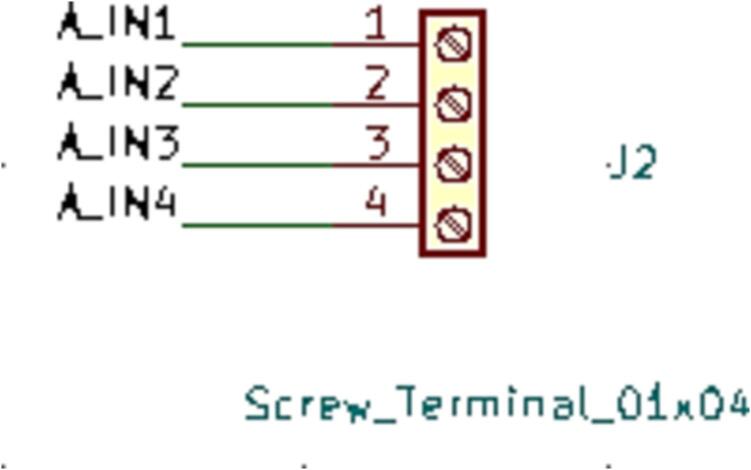
Analog input terminal.

**Fig. 29:**
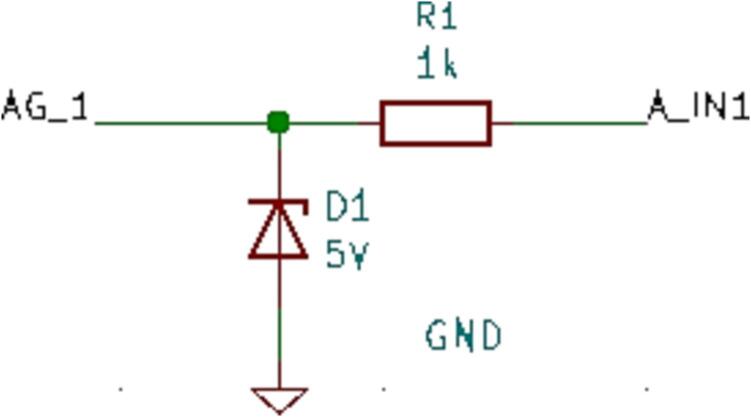
Analog feedback path for each channel.

**Fig. 30:**
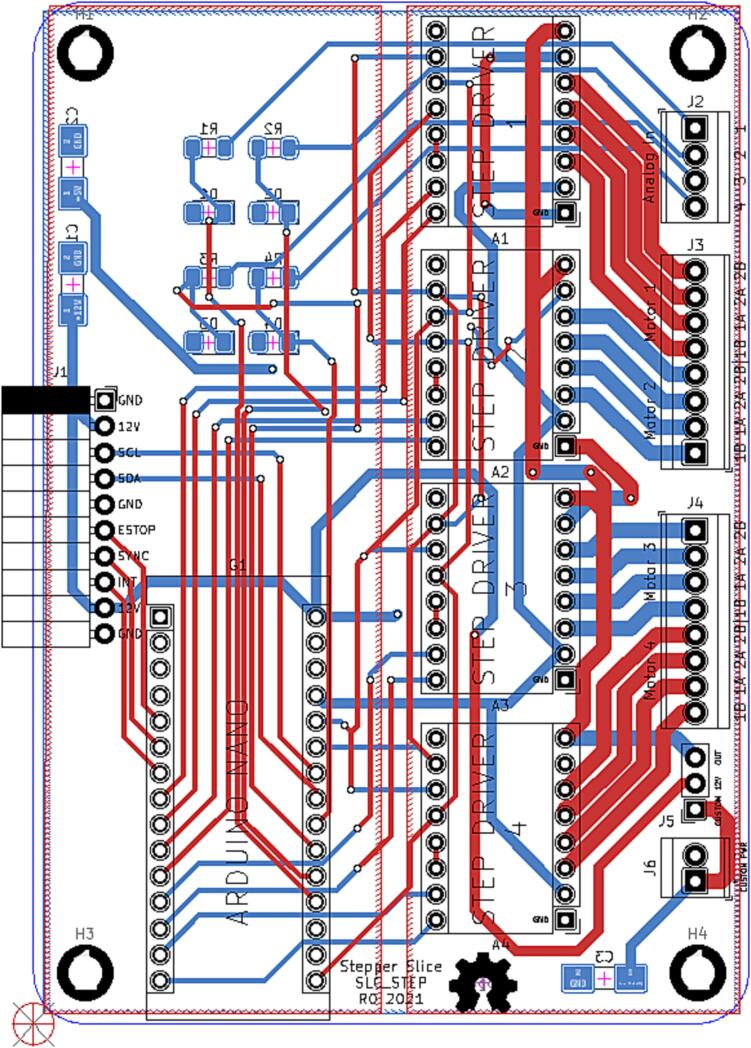
The routed circuit board for SLC_STEP.

SLC_STEP consists of four separately controllable stepper motor control channels (Fig. 25) driven by Pololu A4988 drivers [58] (Fig. 26), common in 3-D printing applications and easily replaceable. There is a port for custom power input (Fig. 27). The step size, direction, speed, acceleration, and much more can be electronically controlled. Analog feedback paths are included (Fig. 28, Fig. 29). Potential applications include: 3-D printer control (and other CNC machines) [32], [59] and electro-mechanical system control [60]. The circuit board layout for the slice is shown in Fig. 30.

The following functionality must be validated for the SLC_STEP:

• Accuracy
• Ability to read in external commands and adjust accordingly.
• Ability to export data quickly and accurately.
• Ability to operate indefinitely and predictably.

Procedure.

Reading Commands.

For slice-only use:

Connect Slice to personal computer and open the Arduino IDE.
Open the Serial Monitor and attempt to transmit a command in proper form.
Verify the system responds properly to all commands that are sent to it. (Peripheral devices may need to be connected to see effects).

For system use:

Insert Slice into back plane.
Command the back plane to send a command to the slice.
Verify the system responds properly to all commands that are sent to it. (Peripheral devices may need to be connected to see effects).

## Exporting data

### Indefinite / Predictability test

#### SLC_THRM 4 Channel Type-K thermocouple Reader

**Fig. 31:**
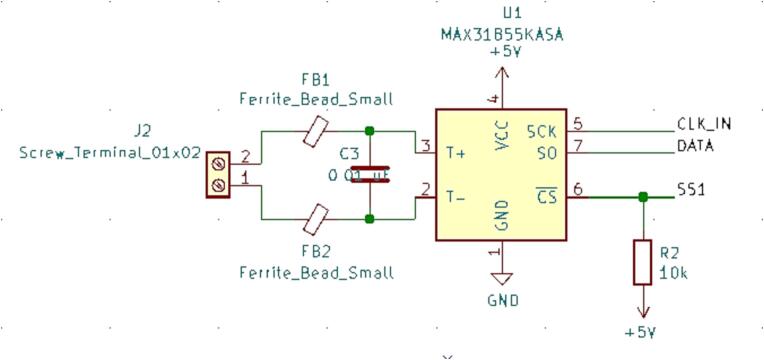
Common thermocouple channel circuitry.

**Fig. 32:**
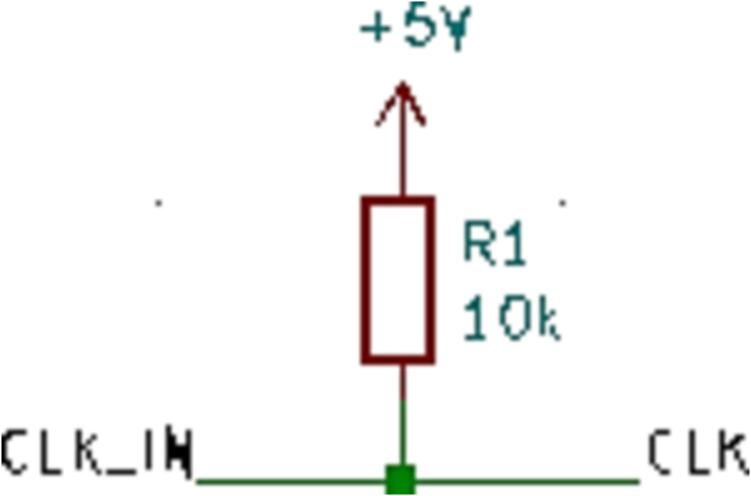
Common SPI Clock Pull-Up.

**Fig. 33:**
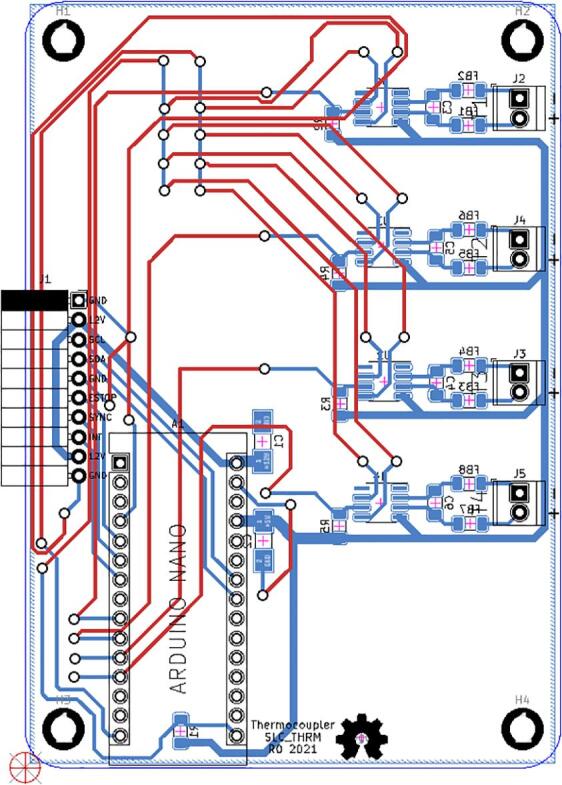
The routed circuit board for SLC_THRM.

SLC_THRM consists of four MAX31855 [61] type-K thermocouple reading chips connected to a thermocouple port, with filtering circuitry in between for smooth reading (Fig. 31). These chips are read by the Arduino via SPI (Fig. 32). Potential applications include closed loop heater control [62] and automatic heat-based alarm system [63]. The circuit board layout for the slice is shown in Fig. 33.

The following functionality must be validated for the SLC_THRM:

• Accuracy
• Ability to read in external commands and adjust accordingly.
• Ability to export data quickly and accurately.
• Ability to operate indefinitely and predictably.

Procedure.

Reading Commands.

For slice-only use:

Connect Slice to personal computer and open the Arduino IDE.
Open the Serial Monitor and attempt to transmit a command in proper form.
Verify the system responds properly to all commands that are sent to it. (Peripheral devices may need to be connected to see effects).

For system use:

Insert Slice into back plane.
Command the back plane to send a command to the slice.
Verify the system responds properly to all commands that are sent to it. (Peripheral devices may need to be connected to see effects).

Exporting Data.

Indefinite / Predictability Test.

#### SLC_USBP 2 Channel USB Port

**Fig. 34:**
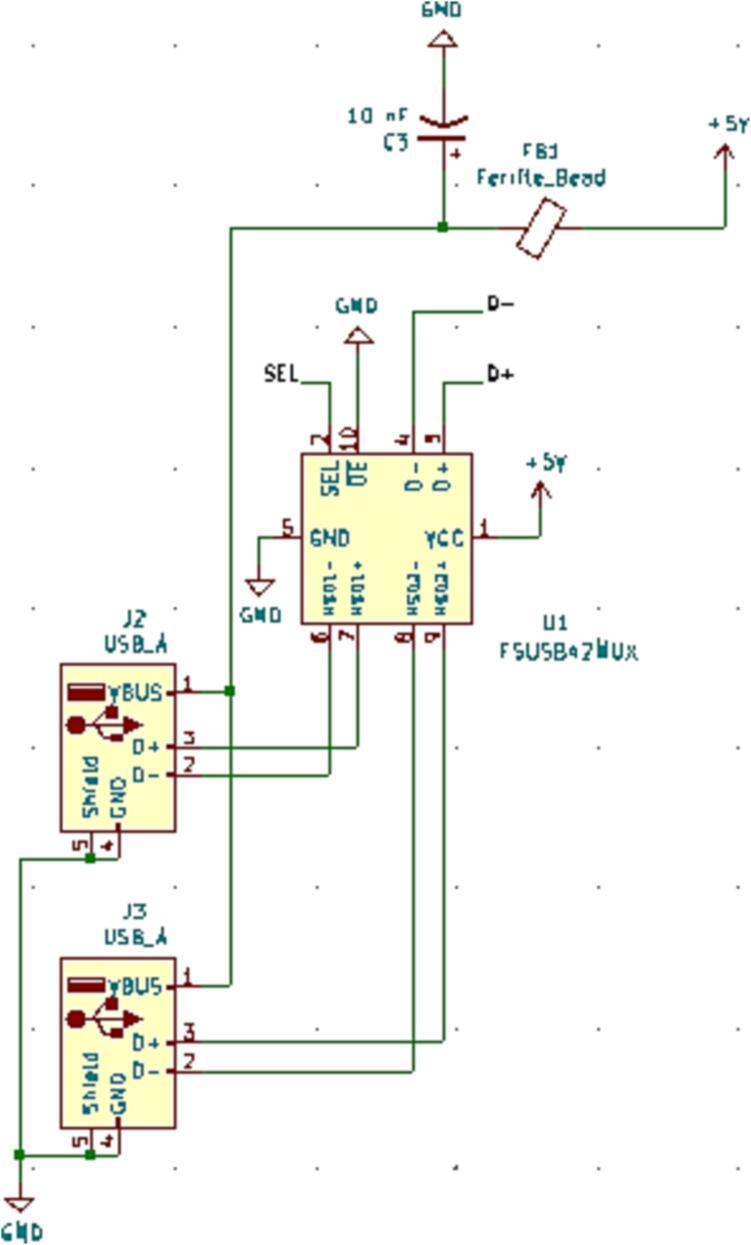
USB ports connected to multiplexer.

**Fig. 35:**
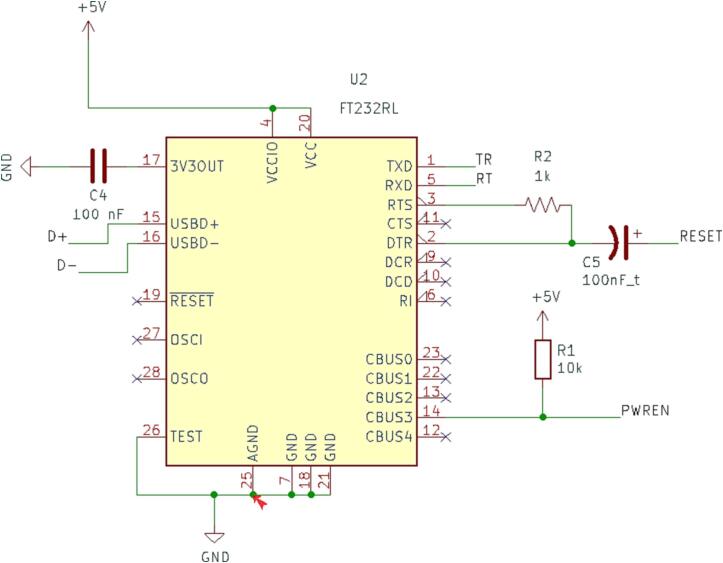
FT232 USB Signal Reader.

**Fig. 36:**
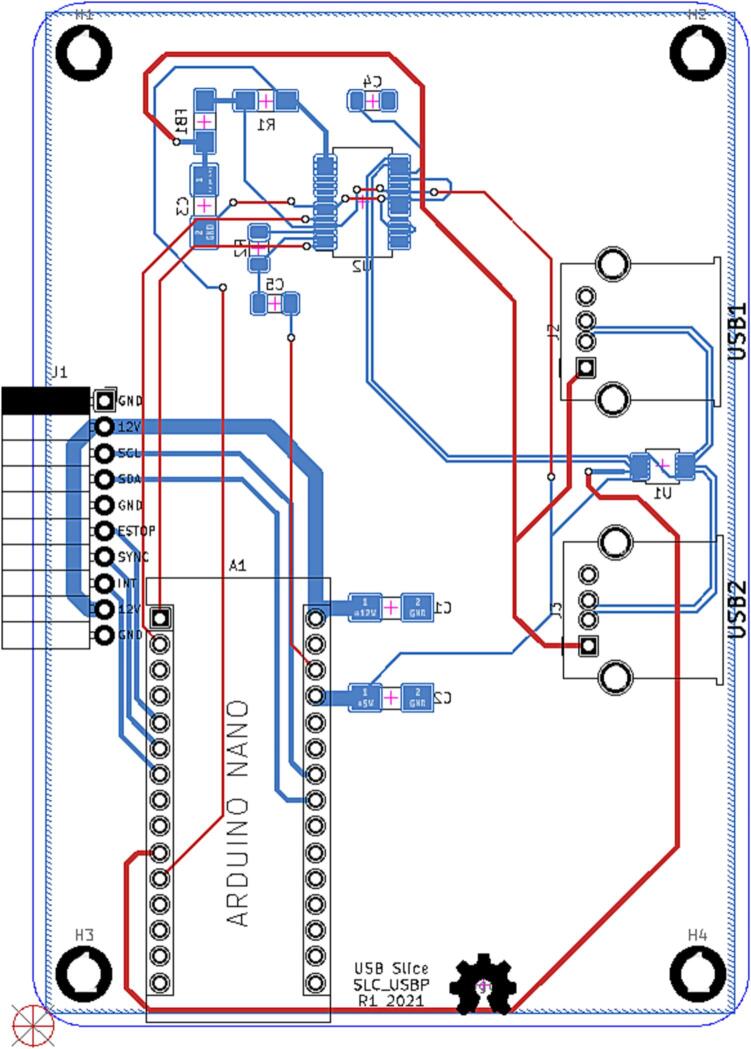
The routed circuit board for SLC_USBP.

SLC_USBP Consists of 2 generic USB A input slots connected to an electrically controlled switch designed for USB port signal switching (Fig. 34). This then connects to an FT232 [64] chip to convert the USB signals to Arduino-readable SPI format (Fig. 35). Potential applications include External device communication and data collection. The circuit board layout for the slice is shown in Fig. 36.

The following functionality must be validated for the SLC_USBP:

Reading Commands.

For slice-only use:

Connect Slice to personal computer and open the Arduino IDE.
Open the Serial Monitor and attempt to transmit a command in proper form.
Verify the system responds properly to all commands that are sent to it. (Peripheral devices may need to be connected to see effects).

For system use:

Insert Slice into back plane.
Command the back plane to send a command to the slice.
Verify the system responds properly to all commands that are sent to it. (Peripheral devices may need to be connected to see effects).

## Design files summary

Each slice and loaf have the following primary design files:

**Table:** 

**Design file name**	**File type**	**Open source license**	**Location of the file**
Firmware/firmware.ino	.ino Arduino Code	GPL 3.0	https://osf.io/742ez/
Gerbers	Gerber board files	CERN OHL-S 2.0	https://osf.io/742ez/
Hardware/Bread_Slice.pro	KiCAD Project	CERN OHL-S 2.0	https://osf.io/742ez/
Hardware/Bread_Slice.sch	KiCAD Schematic	CERN OHL-S 2.0	https://osf.io/742ez/
Hardware/Bread_Slice.kicad_pcb	KiCAD PCB	CERN OHL-S 2.0	https://osf.io/742ez/
Mecahnical/top.stl	STL	CERN OHL-S 2.0	https://osf.io/742ez/
Mecahnical/bottom.stl	STL	CERN OHL-S 2.0	https://osf.io/742ez/

• Firmware/firmware.ino: the default firmware to be programmed onto the slice / loaf
• Gerbers: a folder which contains all gerber files necessary for construction of the circuit board
• Hardware/Bread_Slice.pro: The main project file for the electrical design
• Hardware/Bread_Slice.sch: The schematic of the electrical design
• Hardware/Bread_Slice.kicad_pcb: The board routing for the slice
• Mecahnical/top.stl: The Lid of the enclosure
• Mecahnical/bottom.stl: The base of the enclosure

## Bill of materials summary

SLC_LVAI: Amplified Analog Input Slice [SLC_LVAI].

**Table:** 

**Designator**	**Component**	**Number**	**Cost per unit -currency**	**Total cost -currency**	**Source of materials**
A1	Arduino Nano	1	4.30	4.30	https://www.amazon.com/dp/B08Z7HQ2B6/
C1 – C2	CAP TANT 10UF 10% 25 V 2312	2	0.72	1.44	https://www.digikey.com/short/7vz343n9
C3 – C14	CAP TANT 1UF 10% 25 V 1206	12	0.51	6.12	https://www.digikey.com/short/fzn5wb0d
J1	01x10 Female Header Pins	1	0.79	0.79	https://www.digikey.com/short/23bfwwjh
J2 – J5	TERM BLK 2POS SIDE ENTRY 5MM PCB	4	0.47	1.88	https://www.digikey.com/short/3nv7nt34
R1 – R42	RES 1 K OHM 1% 1/4W 1206	42	0.07	2.94	https://www.digikey.com/short/mbw4qq78
RV1 – RV12	TRIMMER 10 K OHM 0.5 W PC PIN TOP	12	1.79	21.48	https://www.digikey.com/short/w8rr4qdm
U1 – U6	IC OPAMP GP 4 CIRCUIT 14DIP	6	0.063	0.378	https://www.digikey.com/short/5mzc8t8t
PCB	Circuit Board	1	1.29	1.29	https://pcbshopper.com/

SLC_AAFT: Audio Analysis / Fourier Transform [SLC_AAFT].

**Table:** 

**Designator**	**Component**	**Number**	**Cost per unit -currency**	**Total cost -currency**	**Source of materials**
U1-2	4 Stage Amplifier Chip	2	0.44	0.88	https://www.digikey.com/en/products/detail/microchip-technology/MCP6004T-I-ST/523078
J2	Aux Cord Jack	1	1.18	1.18	https://www.digikey.com/en/products/detail/cui-devices/SJ1-3533NG/738701
RV1-4	10 k 12 Turn Trim Pot	4	4.16	16.64	https://www.digikey.com/en/products/detail/bourns-inc/3266 W-1-103LF/1087907
R26	1206 Packaging 360 Ω Resistor	1	0.10	0.10	https://www.digikey.com/en/products/detail/yageo/AC1206FR-07360RL/5897483
R24-25	1206 Packaging 450 Ω Resistor	2	0.10	0.20	https://www.digikey.com/en/products/detail/yageo/RC1206FR-07453RL/728924
R22-23	1206 Packaging 600 Ω Resistor	2	0.10	0.20	https://www.digikey.com/en/products/detail/yageo/RC1206FR-07604RL/729038
R20-21	1206 Packaging 900 Ω Resistor	2	0.10	0.20	https://www.digikey.com/en/products/detail/yageo/RC1206FR-07909RL/729166
R18-19	1206 Packaging 1.8 kΩ Resistor	2	0.10	0.20	https://www.digikey.com/en/products/detail/yageo/RC1206JR-071K8L/729204
R17	1206 Packaging 400 Ω Resistor	1	0.10	0.10	https://www.digikey.com/en/products/detail/yageo/RT1206BRD07400RL/5936956
R13-16	1206 Packaging 500 Ω Resistor	4	0.10	0.40	https://www.digikey.com/en/products/detail/yageo/RC1206FR-07499RL/728944
R1-12	1206 Packaging 1 kΩ Resistor	12	0.10	1.20	https://www.digikey.com/en/products/detail/yageo/RC1206FR-071KL/728387
J3	Quarter Inch Jack	1	1.58	1.58	https://www.mouser.com/ProductDetail/Neutrik/NMJ6HCD2?qs=ZNZTMDotSRlKYl2qFVJbQA%3D%3D
C4-12	1206 Packaging 0.1 µF Capacitor	9	0.16	1.44	https://www.digikey.com/en/products/detail/yageo/CC1206KRX7R8BB104/5884627
C3	1206 Packaging 10 µF Capacitor	1	0.66	0.66	https://www.digikey.com/en/products/detail/yageo/CC1206KKX5R6BB106/5195344
C1-2	1206 Packaging 10 µF Capacitor	2	0.64	1.28	https://www.digikey.com/short/qfrbqfhd
A1	Arduino Nano	1	4.30	4.30	https://www.amazon.com/dp/B08Z7HQ2B6/
J1	01x10 Female Header Pins	1	0.79	0.79	https://www.digikey.com/short/23bfwwjh
PCB	Circuit Board	1	1.29	1.29	https://pcbshopper.com/

SLC_CR10: +/- 10A Current Sensor [SLC_CR10].

**Table:** 

**Designator**	**Component**	**Number**	**Cost per unit -currency**	**Total cost -currency**	**Source of materials**
U1-4	10A Current Sensor Chip	4	5.68	22.72	https://www.digikey.com/en/products/detail/allegro-microsystems/ACS723KMATR-10AB-T/5225374
J2-J5	1/10″ Pitch 01x02 Screw Terminals	4	1.27	5.08	https://www.digikey.com/en/products/detail/te-connectivity-amp-connectors/282834-2/1150135
C1-2	1206 Packaging 10 µF Capacitor	2	0.64	1.28	https://www.digikey.com/short/qfrbqfhd
A1	Arduino Nano	1	4.30	4.30	https://www.amazon.com/dp/B08Z7HQ2B6/
J1	01x10 Female Header Pins	1	0.79	0.79	https://www.digikey.com/short/23bfwwjh
PCB	Circuit Board	1	1.29	1.29	https://pcbshopper.com/

SLC_RLAY: 4 Channel Relay Controller [SLC_RLAY].

**Table:** 

**Designator**	**Component**	**Number**	**Cost per unit -currency**	**Total cost -currency**	**Source of materials**
U2,U4,U3,U1	Optocouplers	4	0.47	1.88	https://www.digikey.ca/en/products/detail/everlight-electronics-co-ltd/EL3H7-B-TA-VG/2675688
RV4,RV3,RV2,RV1	10 k Trimmer Potentiometer	4	5.59	22.36	https://www.digikey.ca/en/products/detail/bourns-inc/3266 W-1-103LF/1087907
R4,R3,R2,R1	1 k 1206 Packaging Resistor	4	6.21	24.84	https://www.digikey.ca/en/products/detail/stackpole-electronics-inc/RMCF1206FT1K00/1759616
K4,K3,K2,K1	G5Q1 SPDT Relay	4	1.66	6.64	https://www.digikey.ca/en/products/detail/omron-electronics-inc-emc-div/G5Q-1-DC5/1815726
J6	01x04 1/10″ Pitch Terminal Block	1	2.01	2.01	https://www.digikey.ca/en/products/detail/on-shore-technology-inc/OSTVN04A150/1588864
J5,J4,J3,J2	01x03 1/10″ Pitch Terminal Block	4	1.51	6.04	https://www.digikey.ca/en/products/detail/on-shore-technology-inc/OSTVN03A150/1588863
D8,D7,D6,D5	5.1 V Zener Diode	4	0.32	1.28	https://www.digikey.ca/en/products/detail/diodes-incorporated/BZT52C15-7-F/755469
D4,D3,D2,D1	Standard THT Diode	4	0.60	2.40	https://www.digikey.ca/en/products/detail/kyocera-avx/SD1206S020S1R0/3749491
C2,C1	10 µF 1206 Packaging Ceramic Capacitor	4	0.81	1.62	https://www.digikey.ca/short/qfrbqfhd
A1	Arduino Nano	1	9.58	9.58	https://www.digikey.ca/en/products/detail/arduino/A000005/2638989
J1	01x10 Female Header Pins	1	1.10	1.10	https://www.digikey.ca/en/products/detail/sullins-connector-solutions/PPPC101LGBN-RC/775943
PCB	Circuit Board	1	1.29	1.29	https://pcbshopper.com/

SLC_STEP: 4 Channel Stepper Motor Controller [SLC_STEP].

**Table:** 

**Designator**	**Component**	**Number**	**Cost per unit -currency**	**Total cost -currency**	**Source of materials**
J5	01x03 1/10″ Pitch Male Connector Pins	1	0.27	0.27	https://www.digikey.com/short/jv0b95z0
C1-3	1206 Packaging 10 µF Capacitor	3	0.64	1.92	https://www.digikey.com/short/qfrbqfhd
J6	01x02 1/10″ Pitch Terminal Block	1	1.27	1.27	https://www.digikey.com/short/0v8hf4h8
R1-4	1 k 1206 Packaging Resistor	4	0.10	0.40	https://www.digikey.com/short/wp8502p7
J3-4	01x08 1/10″ Pitch Terminal Block	2	6.45	12.90	https://www.digikey.com/short/mqn9fd88
J2	01x04 1/10″ Pitch Terminal Block	1	2.75	2.75	https://www.digikey.com/short/tv7tzq1r
D1-4	5.1 V Zener Diode	4	0.22	0.88	https://www.digikey.com/short/z0f0ztp2
A1-4	Pololu A4988 Stepper Driver	4	5.95	23.8	https://www.pololu.com/category/156/a4988-stepper-motor-driver-carriers
G1	Arduino Nano	1	4.30	4.30	https://www.amazon.com/dp/B08Z7HQ2B6/
J1	01x10 Female Header Pins	1	0.79	0.79	https://www.digikey.com/short/23bfwwjh

SLC_THRM: 4 Channel Type-K Thermocouple Reader [SLC_THRM].

**Table:** 

**Designator**	**Component**	**Number**	**Cost per unit -currency**	**Total cost -currency**	**Source of materials**
R1-R5	RES 10 K OHM 1% 1/2W 1206	4	0.10	0.40	https://www.digikey.com/short/p82bnb9p
U1-U4	MAX31855KASA + T	4	7.04	28.16	https://www.digikey.com/short/rbbt74n5
J2-J5	TERM BLK 2POS SIDE ENTRY 5MM PCB	4	0.47	1.88	https://www.digikey.com/short/3nv7nt34
FB1-FB8	FERRITE BEAD 1206 1LN	8	0.17	1.36	https://www.digikey.com/short/709wpzrb
C3-C6	CAP CER 10000PF 50 V X7R 1206	4	0.29	1.16	https://www.digikey.com/short/9d02ztwz
C1-C2	CAP TANT 10UF 10% 25 V 2312	2	0.72	1.44	https://www.digikey.com/short/7vz343n9
A1	Arduino Nano	1	4.30	4.30	https://www.amazon.com/dp/B08Z7HQ2B6/
J1	01x10 Female Header Pins	1	0.79	0.79	https://www.digikey.com/short/23bfwwjh
PCB	Circuit Board	1	1.29	1.29	https://pcbshopper.com/

SLC_USBP: 2 Channel USB Port [SLC_USBP].

**Table:** 

**Designator**	**Component**	**Number**	**Cost per unit -currency**	**Total cost -currency**	**Source of materials**
J2 -J3	CONN RCPT TYPEA 4POS R/A	2	2.52	5.06	https://www.digikey.ca/en/products/detail/molex/0676430910/917619
C1 – C2	CAP TANT 10UF 10% 25 V 2312	2	0.81	1.62	https://www.digikey.ca/short/qfrbqfhd
A1	Arduino Nano	1	9.58	9.58	https://www.amazon.ca/KeeYees-Module-ATmega328P-CH340G-Arduino/dp/B0816SGKHH/
J1	01x10 Female Header Pins	1	1.10	1.10	https://www.digikey.ca/en/products/detail/sullins-connector-solutions/PPPC101LGBN-RC/775943
U1	IC USB SWITCH DPDT 10MSOP	1	1.46	1.46	https://www.digikey.ca/en/products/detail/onsemi/FSUSB42MUX/2036916
U2	IC USB FS SERIAL UART 28-SSOP	1	46.95	6.95	https://www.digikey.ca/en/products/detail/ftdi-future-technology-devices-international-ltd/FT232RL-REEL/1836385
PCB	Circuit Board	1	1.29	1.29	https://pcbshopper.com/

LOAF_X08: 8 Channel Loaf Backplane [LOAF_X08].

**Table:** 

**Designator**	**Component**	**Number**	**Cost per unit -currency**	**Total cost -currency**	**Source of materials**
C1, C2	10UF_T	2	0.81	1.62	https://www.digikey.ca/short/qfrbqfhd
A1	ARDUINO_NANO_V3.X	1	9.58	9.58	https://www.amazon.ca/KeeYees-Module-ATmega328P-CH340G-Arduino/dp/B0816SGKHH/
J1, J2, J3, J4, J5, J6, J7, J8	CONN_01X10_MALE	8	0.25	2.00	https://www.digikey.ca/en/products/detail/adam-tech/PH1-10-UA/9830653
J11, J12	CONN_01X06_FEMALE	2	0.72	1.44	https://www.digikey.ca/en/products/detail/sullins-connector-solutions/PPTC061LFBN-RC/810145
J9	SCREW_TERMINAL_01X02	1	0.68	0.68	https://www.digikey.ca/short/q7wbhd
J10	CONN_01X03_MALE	1	0.14	0.14	https://www.digikey.ca/en/products/detail/adam-tech/PH1-03-UA/9830289
PCB	Circuit Board	1	2.20	2.20	https://pcbshopper.com/

Slice Casing.

**Table:** 

**Component**	**Number**	**Cost per unit -currency**	**Total cost -currency**	**Source of materials**
M3x20 Machine Screw	4	$0.21	$0.84	https://www.amazon.ca/XunLiu-Grade-Alloy-Socket-Screws/dp/B07Q1CRMSW/ref=sr_1_5
M3x10 Machine Screw	4	$0.16	$0.64	https://www.amazon.ca/XunLiu-Grade-Alloy-Socket-Screws/dp/B07PWXN9JR/ref = sr_1_1_sspa
Hex Standoff Threaded M3 Aluminum 0.394″ (10.00 mm)	4	$0.64	$2.56	https://www.digikey.ca/en/products/detail/keystone-electronics/24433/1532165

Loaf Casing.

**Table:** 

**Component**	**Number**	**Cost per unit -currency**	**Total cost -currency**	**Source of materials**
M3x10 Machine Screw	4	$0.71	$2.84	https://www.amazon.ca/XunLiu-Grade-Alloy-Socket-Screws/dp/B07PWXN9JR/ref=sr_1_1_sspa
M3 Nuts	4	$0.08	$0.32	https://www.amazon.ca/Sowaka-Stainless-Silver-Female-Fastener/dp/B09J4C4MLH/ref = sr_1_5

## Build instructions

### General Component construction and programming

When building a component from the BREAD system, first download the entire folder structure from the relevant repository. Order all of the components listed in the BOM.csv file. In cases where the routing is performed on a single layer, the board can either be milled [32] or etched [65]. For more advanced boards (2 layers, or fine-pitched components), the board can be ordered from a commercial manufacturer. Either of the two steps require reference to the files stored in the gerbers directory.

**Fig. 37:**
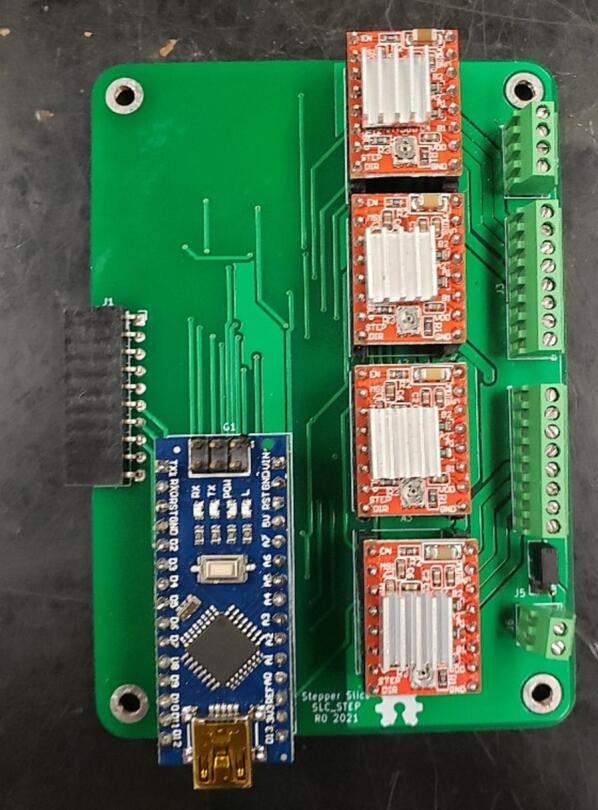
An example assembled slice, the SLC_STEP.

Most of the components are through hole and thus can easily be soldered using a standard fine-tipped soldering iron. SLC_STEP is assembled as an example in Fig. 37.

**Fig. 38:**
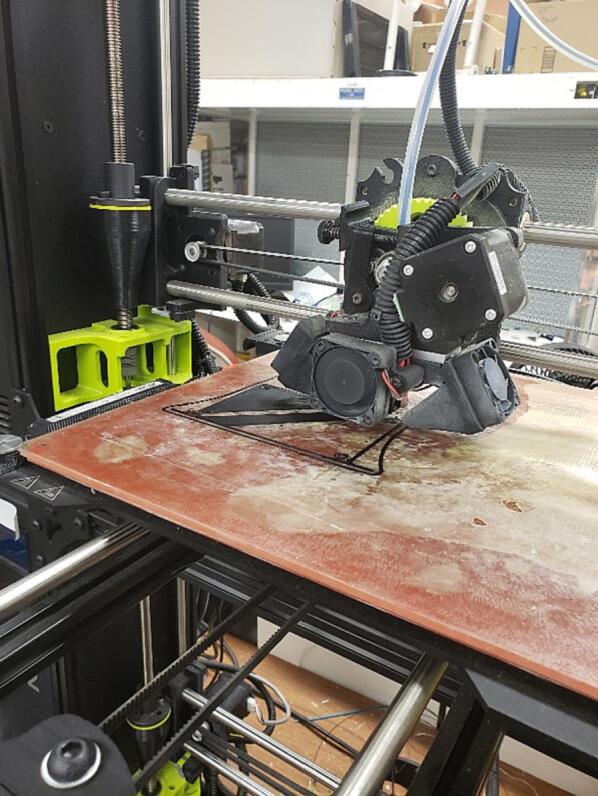
3-D Printing the Case.

The slice or loaf casing can be 3-D printed out of any available rigid material, with solid infill, and a layer height under 0.5 mm (Fig. 38).

**Fig. 39:**
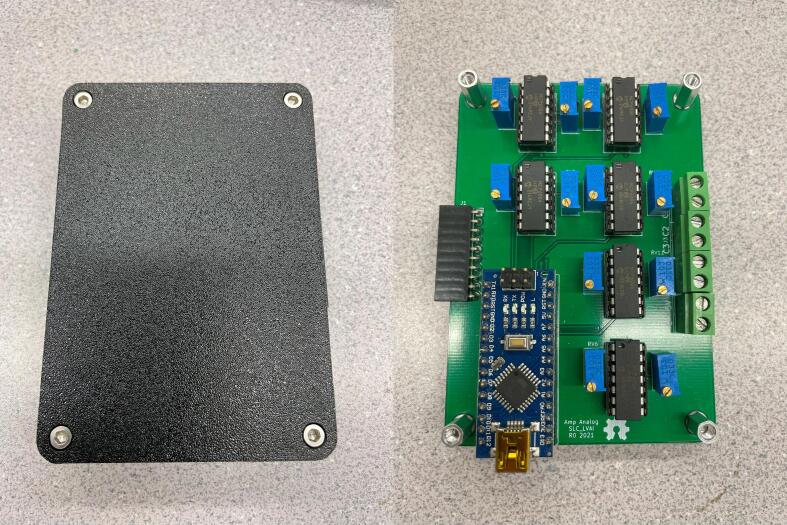
Bottom Slice Case Installed.

Install the bottom case part with M3x10 screws and hex standoffs (Fig. 39).

**Fig. 40:**
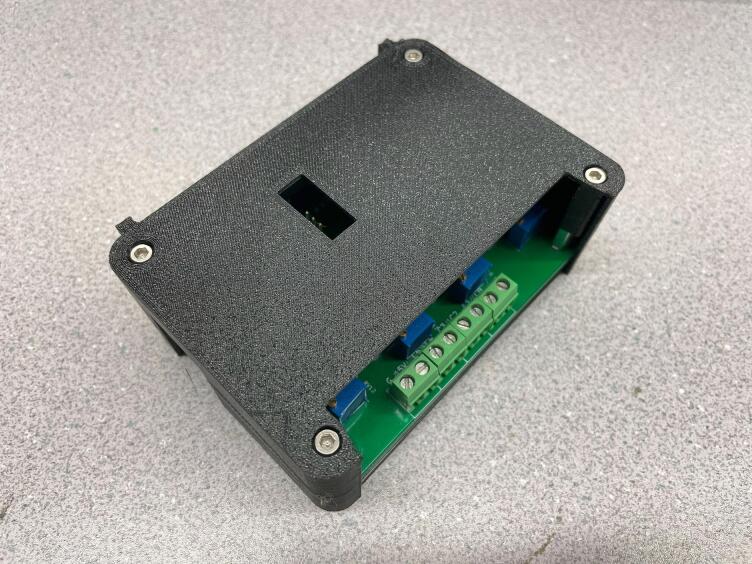
A fully assembled slice.

If necessary, adjust jumpers to the desired position on the board, then install the board and the top cover, using M3x20 screws (Fig. 40).

**Fig. 41:**
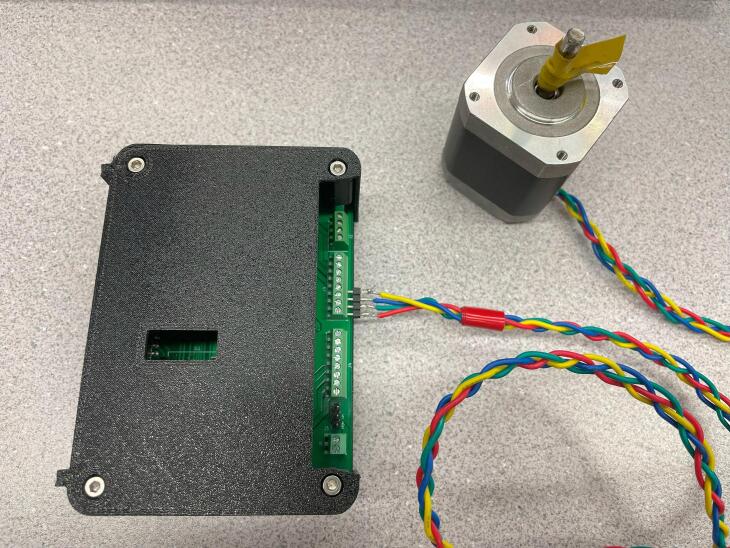
A slice with its relevant peripheral (stepper motor) installed.

In the case where slices are being placed in a loaf, it can be advantageous to wire all peripherals before placing the slice into the loaf as shown with the stepper motor in Fig. 41.

After the slice has been assembled, the Arduino Nano can be programmed with the default firmware in the firmware folder. Open the firmware.ino file in the Arduino IDE [13] and connect the device to the programming computer via a USB cable. Customization of the firmware is supported and encouraged, but it is recommended that the command parsing and communication functions remain unaltered.

### Emergency Stop wiring

**Fig. 42:**
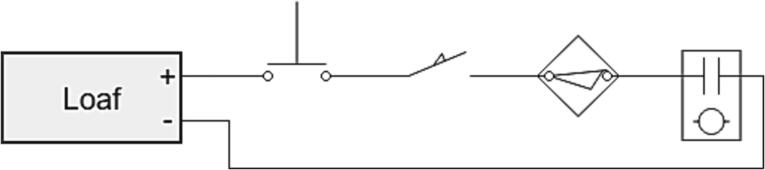
An example safety interlock chain consisting of a push button, limit switch, proximity switch, and a relay contact.

Each loaf has a set of contacts broken out for an emergency stop interlock chain (Fig. 42). Specifically, on the LOAF_X08, the contacts are broken out on J1. The interlock chain can contain any contact-based device such as push buttons, limit switches, proximity switches, and relay contacts. The devices must be wired such that the “open state” indicates the device should be in an E-Stop State. If the E-Stop feature is not being used, these contacts should be shorted together.

### Example System: Metal 3D printer Monitor

The example configuration for monitoring the welding process for a metal 3-D printer utilizes 1 loaf (LOAF_X08), an analog measurement slice (SLC_LVAI), and a thermocouple measurement slice (SLC_THRM). The slices should be programmed with the default firmware. SLC_LVAI should be placed in the Slice 1 slot, and SLC_THRM should be placed in the Slice 2 slot.

**Fig. 43:**
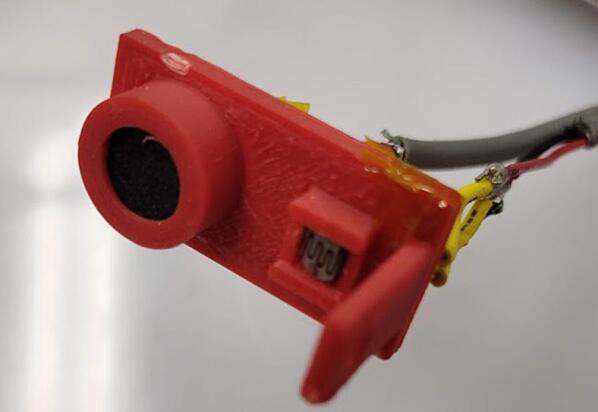
The Light and Sound measurement fixture.

**Fig. 44:**
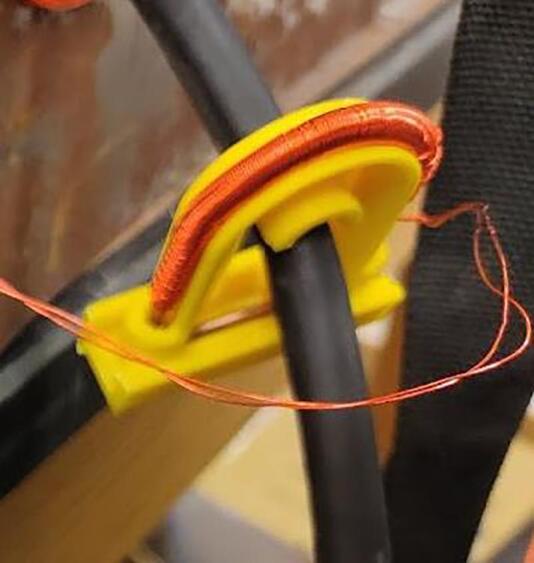
A custom current transformer placed around the ground wire of the welder.

The piezoelectric microphone and photoresistor should be linked to suitably long twisted shielded pair wires to shield the signals from any potential interference due to the welding process. The sensors are placed in a 3-D printed carrier (Fig. 43) and mounted to the end effector of the 3-D printer. The sensors are then linked into input channels on the SLC_LVAI. Additionally, a custom current transformer (Fig. 44), which can be manufactured using an open source winder [66] is placed around the ground wire of the welder (note that any current transformer can be substituted for this sensor). The transformer is also wired to SLC_LVAI. Finally, 4 type-k thermocouple probes are attached underneath the build substrate, electrically insulated by a thin piece of Kapton tape.

A worse-case scenario weld should be initialized (Turn off the gas and raise the tungsten electrode to ∼ 3 mm), and then the offset and gain of each analog channel should be adjusted to fully utilize the resolution of the ADC.

The loaf output can be monitored via the Arduino Serial Monitor (or equivalent) at 250,000 baud.

## Validation and characterization

### Slice characterization

Each of the eight slices has undergone a basic characterization, and the results are listed below in their respective sections. All slices have had basic communication functionality validated (both via the Backplane and via USB).

#### SLC_LVAI: Amplified Analog input slice characterization

**Fig. 45:**
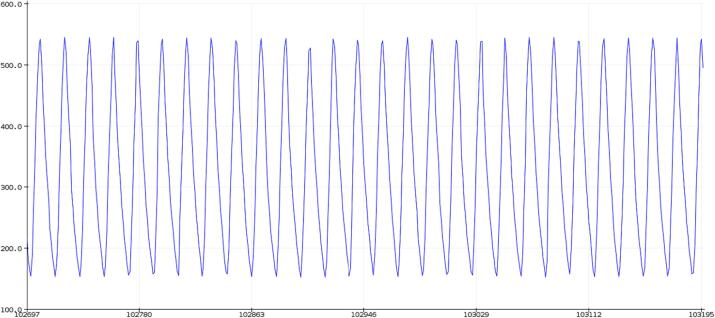
An example Analog reading generated from a signal supply linked to Channel 1 rendered in the Arduino IDE from serial data.

**Fig. 46:**
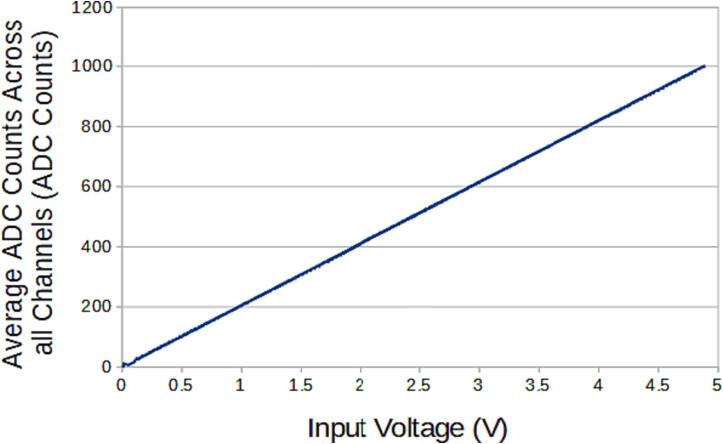
The average response of the channel per input voltage.

**Fig. 47:**
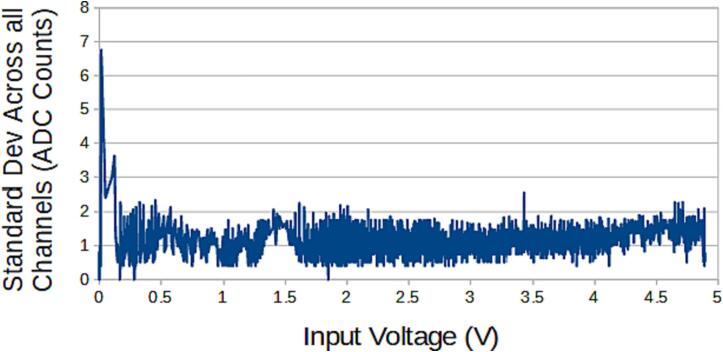
The standard deviation across all channels per voltage input.

The SLC_LVAI is a flexible analog input card, bolstered by it’s hardware gain and offset. Key features are summarized in Table 3. When linked to USB, the slice can act as an effective method for low frequency signal visualization (Fig. 45). The channels act linearly (Fig. 46) and exhibit a low deviation from channel to channel (Fig. 47).

**Table 3:** A Table summarizing the key features of the Analog Slice.

**Parameter**	**Value (or Range)**
Allowable Voltage Range	0 V – 5 V
Maximum Frequency	120 Hz
Maximum Gain	20.82 dB
Maximum Offset	5 V

#### SLC_AAFT: Audio analysis / Fourier Transform characterization

**Fig. 48:**
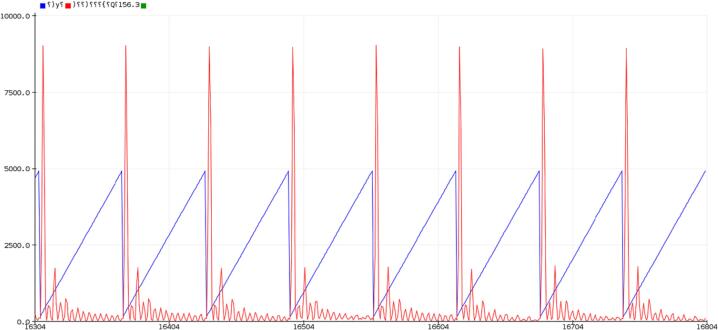
An example FFT reading generated from an electric guitar linked to Channel 1. The chord being played is Middle Cmaj. Red is the FFT Output, and blue is tracking the relevant frequency bin rendered in the Arduino IDE from serial data.

**Fig. 49:**
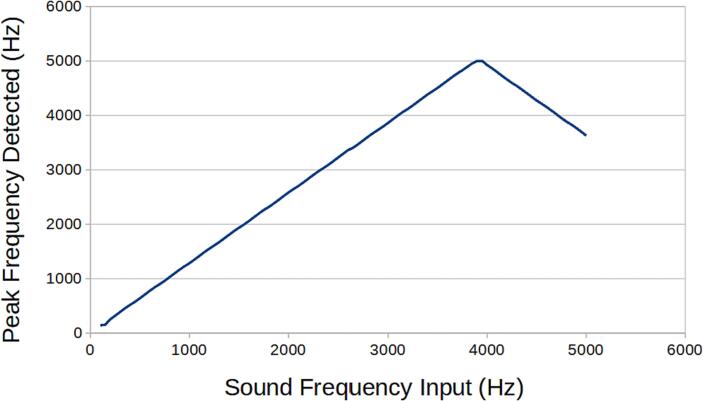
A varying frequency is input into a channel of the AAFT and the peak frequency is detected.

Key features are summarized in Table 4. When connected directly to USB, the SLC_AAFT acts an effective tool for visualizing signal spectra (Fig. 48). When exposed to a frequency, the uncalibrated SLC_AAFT shows linearity up until roughly 3.8 kHz, at which case the measurement becomes aliased (Fig. 49). This experimentally determines the usable range of the ADC to be 0 to 3.5 kHz.

**Table 4:** A Table summarizing the key features of the Audio Slice.

**Parameter**	**Value (or Range)**
Allowable Voltage Range	0 V – 5 V
Maximum Frequency	3.5 kHz
Maximum Gain	20.82 dB
Maximum Offset	5 V

#### SLC_CR10: +/- 10A current sensor characterization

**Fig. 50:**
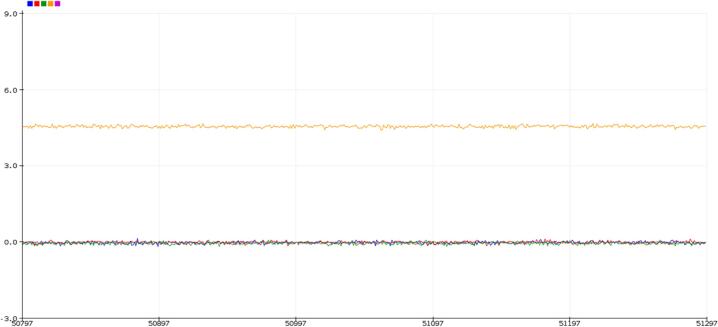
A DC current of 4A is passed through CH1, while the others are left at 0A. Rendered in the Arduino IDE from serial data.

**Fig. 51:**
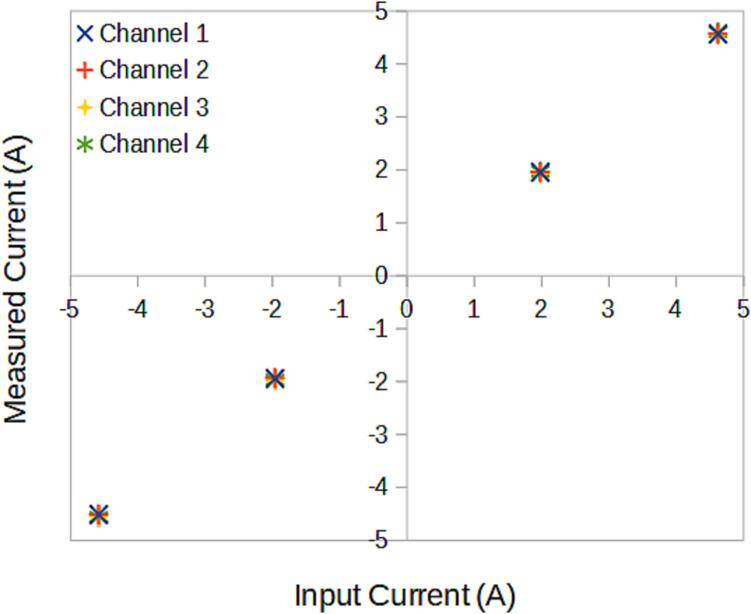
The current across all channels given 4 test loads.

Key features are summarized in Table 5. When connected directly to USB, the SLC_CR10 can visually display up to four current measurements simultaneously (Fig. 50). All four channels respond linearly (Fig. 51) with a standard deviation across all channels of 0.02A.

**Table 5:** A Table summarizing the key features of the Current Slice.

**Parameter**	**Value (or Range)**
Maximum Measurable Current	−10A to 10A
Minimum Measurable	20 mA
Maximum Frequency	100 Hz

#### SLC_RLAY: 4 Channel Relay Controller characterization

SLC_RLAY can be used for switching up to four high current devices and measuring up to four DC voltage channels. Analog inputs were validated by tuning the maximum input to 12VDC and measuring a 5VDC source. Key features are summarized in Table 6.

**Table 6:** A Table summarizing the key features of the Relay Slice.

**Parameter**	**Value (or Range)**
Max Current	10A AC / 5A DC
Max Voltage	125 V AC / 30VDC
Switch Time	5 ms
Max Input Voltage	100VDC
Max Measurement Deviation	+/- 0.13VDC

#### SLC_STEP: 4 Channel Stepper motor Controller characterization

SLC_STEP provides a flexible platform which can control a wide range of bi-polar stepper motors. If the stepper can operate under 2A per coil, and at speeds less than 10,000 steps per second, the SLC_STEP can drive them. This has been validated by running commercial NEMA 17 motors with no errors. Key features are summarized in Table 7.

**Table 7:** A Table summarizing the key features of the Stepper Slice.

**Parameter**	**Value (or Range)**
Max Motor Current	2A per coil
Max Output Rate (Used to determine speed)	10,000 Pulses / second

#### SLC_THRM: 4 Channel Type-K thermocouple Reader characterization

**Fig. 52:**
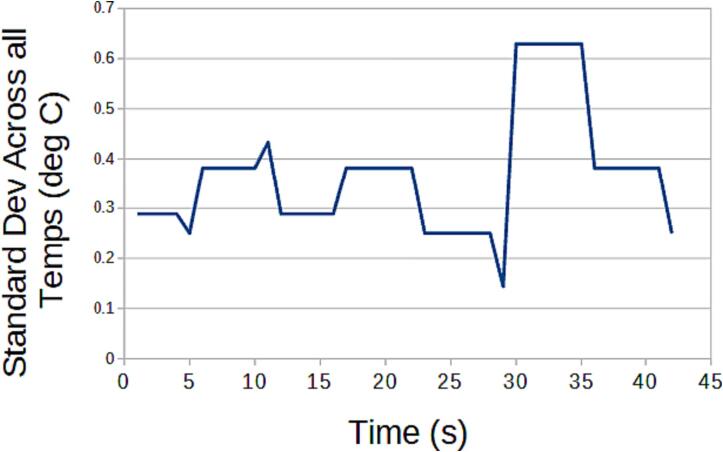
The standard deviation across all channels given a stable room temperature.

Key features are summarized in Table 8. SLC_THRM can read from Type-K thermocouples with little noise (Fig. 52).

**Table 8:** A Table summarizing the key features of the Thermocouple Slice.

**Parameter**	**Value (or Range)**
Temperature Range	−200C to 1260 °C
Minimum Measurable	0.25 °C

#### SLC_USBP: 2 Channel USB Port characterization

**Fig. 53:**
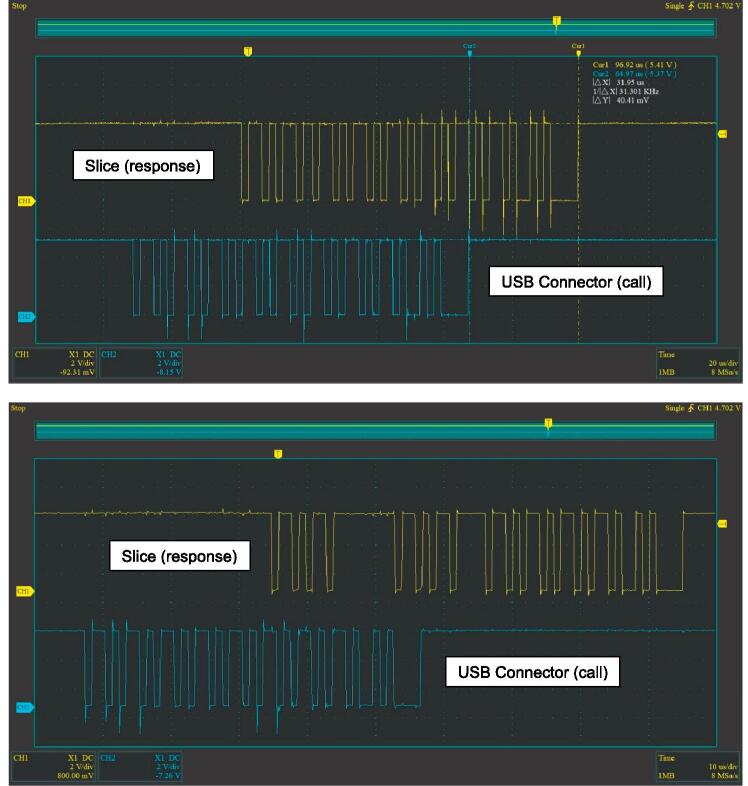
Exchange of four “k” characters in a row over UART (top 0.5Mbps, bottom 1Mbps).

SLC_USBP can connect and communicate with up to two USB devices at a speed compatible with USB 2.0 (or slower). Key features are summarized in Table 9. Upon testing, the maximum achievable data transfer speed was 0.5Mbps. This was tested by sending a single character through the USB connector to the slice, receiving the character, and sending it back through the USB connector. An oscilloscope was used to monitor this exchange. The slice can send up to 64 characters (bytes) in a single transmission; however, at speeds higher than 0.5Mbps, the data becomes depreciated as can be seen with pausing in between pulses (Fig. 53).

**Table 9:** A Table summarizing the key features of the USB Slice.

**Parameter**	**Value (or Range)**
Max Speed	0.5Mbps

### Loaf characterization

The Loaf backplane can communicate with up to eight slices on a single board with a theoretical maximum of 128 slices. Table 10 outlines the key characteristics of the Loaf.

**Table 10:** A Table summarizing the key features of the Loaf backplane.

**Parameter**	**Value (or Range)**
Max Input Voltage	20 V DC
Max Total Current Draw (from all slices)	50A DC

### Example System: TIG-based metal 3D printer Monitor

**Fig. 54:**
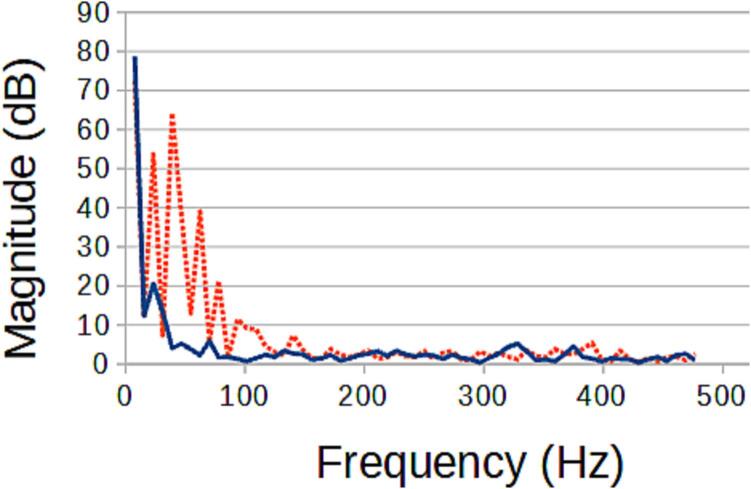
The light frequency spectrum of an ideal weld (blue solid) compared to the frequency spectrum of a flawed weld (red dotted).

**Fig. 55:**
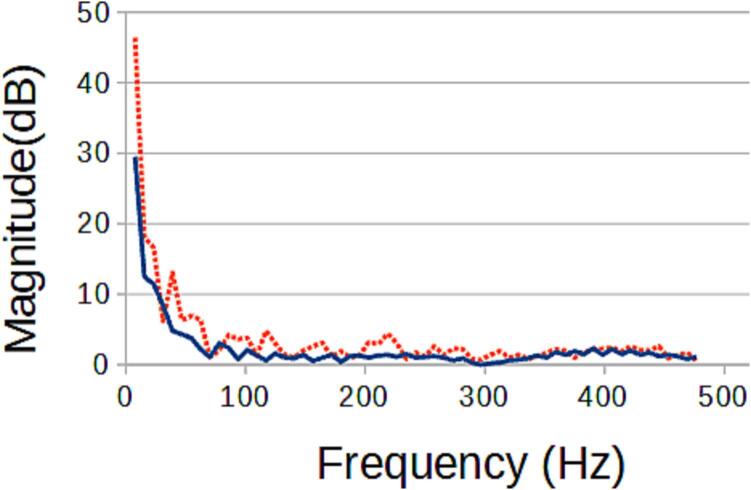
The sound frequency spectrum of an ideal weld (blue solid) compared to the frequency spectrum of a flawed weld (red dotted).

The TIG-bot, an open source TIG-base metal 3-D printer [66], was set to run 2 tests, where a 16 mm long line is extruded, with only the stand-off distance varying (which will directly impact weld quality). As the welding process is DC, there is no significant signal aside from the start up. This binary signal, however, can be used to determine if a start-up was successful. This basic experiment has shown that the target signals are affected by the experimental variable and can now be correlated (Fig. 54, Fig. 55). This information can be used for a multitude of purposes such as close looped control to maintain an ideal weld, or instantaneous weld characterization.

### Economic analysis

The closest commercial alternative to the BREAD system is National Instruments CompactRIO (cRIO) system. Like BREAD, it offers a backplane with several interchangeable cards. The focus of the cRIO system is to work with other industrial automation devices, where the aim of BREAD is to be completely inclusive. As an example, the cRIO system would need a digital output card AND an external motor driver, whereas BREAD features an all-in-one solution.

Five of the slices explored in this paper have near-equivalent counterparts (in base functionality) in the cRIO system, and have been compared in Table 11, Table 12, Table 13, Table 14, Table 15 below:

**Table 11:** A comparison of key features for analog measurement devices.

**Description**	**# Channels**	**Max voltage**	**Resolution**	**Sample Rate**	**Gain Range**	**Offset Range**	**Cost (USD)**
SLC_LVAI	6 Single Ended	5 V	10 Bit	10 kS/S	11	5 V	40.61
NI-9281 [67]	2 Differential	12 V	24 Bit	513 S/S	1	0 V	1369.00
**Cost Reduction (%)**							97.0%

**Table 12:** A comparison of key features for audio measurement.

**Description**	**# Channels**	**Max voltage**	**Resolution**	**Max Freq**	**Gain Range**	**Offset Range**	**Cost (USD)**
SLC_AAFT	2	5 V	10 Bit	3.5 kHz	11	5 V	32.64
NI-9230 [68]	3	30 V	24 Bit	6.4 kHz	1	0 V	755.00
**Cost Reduction (%)**							95.7%

**Table 13:** A comparison of key features for current measurement.

**Description**	**# Channels**	**Current Range**	**Resolution**	**Sample Rate**	**Cost (USD)**
SLC_CR10	4	+-10A	10 bit	10 kS/S	35.46
NI-9227 [69]	4	+-10A	24 Bit	1 kS/S	1599.00
**Cost Reduction (%)**					97.8%

**Table 14:** A comparison of key features for mechanical relay switching.

**Description**	**# Channels**	**Max Voltage**	**Max Current**	**Switch Time**	**Type**	**Cost (USD)**
SLC_RLAY	4	125VAC / 30VDC	10 AAC / 5 ADC	5 mS	Electromechanical	46.81
NI-9482 [70]	4	250VAC / 60VDC	1.5 AAC / 1.5 ADC	15 mS	Electromechanical	249.00
**Cost Reduction (%)**						81.2%

**Table 15:** A comparison of key features for temperature measurement.

**Description**	**# Channels**	**Type Compatibility**	**Resolution**	**Cost (USD)**
SLC_THRM	4	All Standard Types	0.25 °C	40.87
NI-9210 [71]	4	All Standard Types	0.8 °C	465.00
**Cost Reduction (%)**				91.2%

The average cost savings of a BREAD slice compared to an equivalent National Instruments cRIO card is 93%. The highest advantage the National Instruments cRIO system can provide is high measurement resolution due to their 24-bit ADCs. However, BREAD’s open design philosophy allows designs such as the SLC_LVAI to have on-board tuneable gain, effectively increasing resolution for lower voltage ranges. Additionally, if high resolution is required, a new slice can be easily evolved from SLC_LVAI using external ADCs (at an added cost).

### Discussion

There are countless open source circuits that are well executed, yet only serve one set of purposes. On *HardwareX* alone, there are systems for irrigation management [72], colorimetry [73], spectral acquisition [74], [75], and specialized environmental sensing circuits [76], [77]. The aforementioned electronics are a very small fraction of existing and documented designs that could be highly compatible with BREAD. The BREAD system can sustain rapid growth by adapting these existing and open designs into new slices.

Since the BREAD system currently has a significant overlap with the National Instruments cRIO system, there are several applications that could easily be adapted for cost reductions. Specifically, BREAD could be employed as a general-purpose data acquisition system for experiments and measurements [78], [79]. BREAD can be employed for measuring and recording power usage and analysis [80], [81], [82]. BREAD can also be used for industrial automation, including robotics [83] and process control [84]. As BREAD’s slice library grows, the capabilities and permutations create an endlessly flexible and capable choice for an electronics platform.

### Conclusions

For researchers using proprietary DAQ systems for environmental sensing, control electronics for automating experiments, or DAQ systems for power monitoring, to name a few examples, BREAD can be a more customizable, inexpensive, and easy to use alternative. The open source nature of BREAD means that researchers can modify the electronics to precisely meet their needs and easily perform repairs and maintenance as needed. The power of BREAD lies in its customizability, modular design, and the fact that all components can be 3-D printed or purchased from common commercial sources.

### Implications and future work

There are several areas of future work that will expand the utility of the BREAD system. First, to make the BREAD system more accessible to new users, a GUI Interface can be developed. In addition, although seven Slices were demonstrated here the Library of Slices can be expanded to increase the functionality of the BREAD system. One way to do this is to convert existing open source electronic systems over to the BREAD format. Future work can also expand the interrupt capabilities. To enable BREAD to work in computer vision, a Pi Zero Slice can, for example, be developed. For more intensive data processing applications, a more powerful processor could be integrated into each Slice like in STM32 or ESP32 cards compatible with Arduino. Finally, BREAD can be explored as a teaching and learning tool. As there is a substantial number of Slices that still need to be developed this could be an ideal virtual service learning [85], [86], [87] or commissioned assignment [88], [89] for advanced electrical engineering students.

### CRediT authorship contribution statement

**Shane Oberloier:** Conceptualization, Methodology, Software, Data curation, Visualization, Investigation, Validation. **Nicholas G. Whisman:** Software, Data curation, Visualization, Investigation, Validation. **Finn Hafting:** Software, Data curation, Visualization, Investigation, Validation. **Joshua M. Pearce:** Supervision, Writing – original draft, Writing – review & editing.

## Declaration of Competing Interest

The authors declare that they have no known competing financial interests or personal relationships that could have appeared to influence the work reported in this paper.
